# The mammalian mitochondrial epitranscriptome^☆^

**DOI:** 10.1016/j.bbagrm.2018.11.005

**Published:** 2019-03

**Authors:** Pedro Rebelo-Guiomar, Christopher A. Powell, Lindsey Van Haute, Michal Minczuk

**Affiliations:** a MRC Mitochondrial Biology Unit, University of Cambridge, Cambridge, UK; b Graduate Program in Areas of Basic and Applied Biology (GABBA), University of Porto, Porto, Portugal

## Abstract

Correct expression of the mitochondrially-encoded genes is critical for the production of the components of the oxidative phosphorylation machinery. Post-transcriptional modifications of mitochondrial transcripts have been emerging as an important regulatory feature of mitochondrial gene expression. Here we review the current knowledge on how the mammalian mitochondrial epitranscriptome participates in regulating mitochondrial homeostasis. In particular, we focus on the latest breakthroughs made towards understanding the roles of the modified nucleotides in mitochondrially-encoded ribosomal and transfer RNAs, the enzymes responsible for introducing these modifications and on recent transcriptome-wide studies reporting modifications to mitochondrial messenger RNAs. This article is part of a Special Issue entitled: mRNA modifications in gene expression control edited by Dr. Matthias Soller and Dr. Rupert Fray.

## Highlights

• Human mtDNA encodes 13 proteins and all the RNAs necessary for their expression
• Post-transcriptional modifications of RNA, the epitranscriptome, play a regulatory role in mitochondrial gene expression
• Several enzymes involved in the shaping of the mitochondrial epitranscriptome have recently been characterised.
• Our understanding of the extent and nature of mtRNA modifications is rapidly expanding.
• Recent transcriptome-wide studies suggest modifications in mitochondrial mRNAs

## 1 Introduction

### 1.1 Mammalian mitochondrial gene expression: overview

#### 1.1.1 Mammalian mitochondrial genome

Present-day mitochondria originated from an endosymbiotic relationship in which an alpha-proteobacteria-like organism was engulfed by another cell, either by a primitive eukaryotic ancestor or by ancient anaerobic archaebacteria. Since then, mitochondria have almost completely lost their autonomy by gradual horizontal transfer of genetic material to the cell nucleus [1]. As a result, modern Metazoa harbour two genomes: one nuclear (nDNA) and another small, vestigial in mitochondria (mtDNA). Human mtDNA is a double stranded, circular molecule of 16,569 kb [2], with a very compact organization of genetic information. The two strands of mtDNA are designated heavy (H) or light (L) based on their buoyancy in caesium chloride density gradients owing to different nucleotide content. The mammalian mitochondrial genome encodes 2 rRNAs, 22 tRNAs and mRNAs for 13 polypeptides of the oxidative phosphorylation (OxPhos) system. The genes lack introns and the coding sequences are either contiguous or separated only by a few non-coding bases [2]. Furthermore, in some cases, mitochondrial protein coding genes (MT-ATP8/6 and MT-ND4/4L) or mitochondrial tRNA genes (mt-tRNA^Tyr^/mt-tRNA^Cys^) have overlapping regions [2,3].

#### 1.1.2 Mammalian mitochondrial transcription

Transcription of the mammalian mitochondrial genome originates from H-strand (HSP) and L-strand (LSP) promoters located in the major non-coding region (NCR) and produces near entire genome length polycistronic transcripts. Most of the genetic information is transcribed from HSP and includes 14 mt-tRNAs, 2 mt-rRNAs and 12 mt-mRNAs, whereas LSP-driven transcription produces the remaining 8 mt-tRNAs and 1 mt-mRNA, encoding the ND6 subunit. Transcription in human mitochondria is driven by a monomeric, T-odd phage related, RNA polymerase (POLRMT) [4]. In contrast to bacteriophage RNA polymerases that recognise promoter sequences without additional factors, auxiliary proteins are required for efficient transcription initiation by POLRMT. These include mitochondrial transcription factors A (TFAM) and B2 (TFB2M). TFAM is a high mobility group (HMG) motif-containing DNA-binding protein, that, in addition to transcription activation, also has a role in packaging mtDNA [5]. TFB2M functions in mtDNA melting during transcription initiation. It evolved as a product of gene duplication (see below), with its paralogue, TFB1M, being a mitochondrial rRNA methyltransferase (Section 2) [[6], [7], [8], [9], [10]]. According to recent biochemical and structural evidence, TFAM binds to mtDNA and recruits POLRMT to the promoter during the formation of the transcription initiation complex, followed by the modification of the POLRMT structure by TFB2M to melt the promoter [8,11]. The transcription elongation step requires TEFM (transcription elongation factor, mitochondrial) [12]. Recombinant TEFM strongly enhances POLRMT processivity as it stimulates the formation of longer transcripts *in vitro* [13,14], whereas inactivation of the *TEFM* gene in living cells leads to a reduction in promoter-distal transcripts [12]. Recent structural work showed that TEFM forms a ‘sliding clamp’ around the mtDNA downstream of the transcribing POLRMT, thereby enhancing its processivity [11].

#### 1.1.3 Mammalian mitochondrial RNA maturation

The polycistronic transcripts generated from both strands of mtDNA are endonucleolyticaly processed and subsequently matured to generate mt-rRNA, mt-tRNA and mt-mRNA molecules. The mt-tRNA genes separate most of rRNA and protein coding genes, thus cleavage of mt-tRNA releases nascent mt-rRNA and mt-mRNA for subsequent processing [2,15]. The 5′-end processing of mt-tRNAs in the polycistronic transcripts is performed by the mitochondrial RNase P, a heterotrimeric complex and, in contrast to earlier assumptions, lacking the catalytic RNA component [16]. The 3′-ends of mt-tRNAs are cleaved by the RNase Z endonuclease encoded by *ELAC2* [[17], [18], [19]]. Released mt-tRNA molecules are matured by the addition of CCA [20,21] and, in some cases, deadenylation of their 3′-end [22], before being aminoacylated, and matured by introduction of multiple post-transcriptional nucleotide modifications described in detail by this review (Section 3). Similarly, mt-rRNAs released from the precursor transcripts undergo multiple post-transcriptional nucleotide modifications, covered by this review (Section 2). As mammalian mitochondrial genes do not contain introns, maturation of nascent mRNAs is essentially limited to constitutive polyadenylation of the 3′-ends of these molecules, with the exception of the mRNA for MT-ND6, which is not polyadenylated [15]. New evidence suggests that several of mt-mRNA contain modified nucleotides, which is also discussed in this article (Section 4).

Post-transcriptional processing of mitochondrial transcripts, and also initial steps of the mitochondrial ribosome assembly, take place in non-membrane delineated structures known as mitochondrial RNA granules (MRGs) [[23], [24], [25], [26], [27]]. These structures contain newly synthetized mtRNA and a number of RNA-binding proteins responsible for stabilisation, processing, modification, folding, and degradation of mtRNA. MGRs are frequently seen in association with the inner mitochondrial membrane and are proximal to mitochondrial nucleoids (reviewed in [28]).

#### 1.1.4 Mammalian mitochondrial translation

The mammalian mitochondrial translation machinery is specialized for the synthesis of the 13 mitochondrially-encoded subunits of the OxPhos system, which is performed by dedicated mitochondrial ribosomes (mitoribosomes). While a complete set of RNA components of the mitochondrial protein synthesis apparatus is encoded by mtDNA, all proteins required for mitochondrial translation, including mitochondrial ribosomal proteins (MRPs), translation initiation, elongation and termination factors, and aminoacyl-tRNA synthetases are encoded in the cell nucleus and imported into the mitochondrial matrix. The details of the organization and regulation of mitochondrial protein synthesis has been covered by recent reviews [[29], [30], [31]].

### 1.2 The nature and roles of RNA post-transcriptional modifications: overview

RNA modifications are found in all three kingdoms of life, and to date, 171 different types of modifications have been identified in cellular RNA [32]. These modifications expand the chemical properties of the four standard nucleosides and are collectively described as the “epitranscriptome”. With the recent development of transcriptome-wide mapping approaches it has been shown that RNA modifications occur on all types of RNA, although in different frequencies, ranging from up to 17% modified nucleotides in cytosolic eukaryotic tRNA [33] to just sparse, low stoichiometry modifications in nuclear-encoded mRNA [34]. Post-transcriptional RNA modifications range from the addition of a simple group (*e.g.* methyl) to that of complex moieties (*e.g.* carboxymethylaminomethyl). Modifications may also include substitution (*e.g.* uridine to 4-thiouridine), oxidation (*e.g.* 5-methylcytidine to 5-hydroxymethylcytidine), reduction (*e.g.* uridine to dihydrouridine) and isomerization (*e.g.* uridine to pseudouridine). Modifications have been described for all four canonical nucleotides, with the majority of modified bases deriving from uridine. Any given modification can occur at different positions of nucleotides, resulting in different chemical structures and presumably different functions. Modifications of both the nucleobase and the ribose of ribonucleosides have been described.

Chemical modifications can occur at all three edges on a ribonucleoside, with base modifications affecting both the Watson-Crick and Hoogsteen edges, and ribose methylations affecting the sugar edge. Depending on the face where they are present, modifications to nucleobases can have distinct effects on the RNA substrate. For example, in the case of guanine, 1-methylguanine (m^1^G) only affects the Watson-Crick edge of the nucleobase, resulting in a slightly destabilized interaction with cytosine, while 7-methylguanine (m^7^G), which affects the Hoogsteen edge, introduces a positive charge on the nucleobase, strongly stabilising hydrogen bonding [35].

Another type of frequent post-transcriptional RNA modification, methylation at the 2′-hydroxyl group of the ribose moiety (2′-*O*-methylation) of the nucleotide is the most frequent RNA methylation [36]. The 2′-*O*-methylation is important for the folding of rRNA and the fidelity of the ribosome. It has been shown that methylation of the 2′-hydroxyl favours the C3′-*endo* form of the ribose by steric repulsion between the nucleobase and the C2′ methoxy group [37]. For example, this conformation is thought to contribute to efficient codon recognition by tRNAs containing modified pyrimidines in position 32 of the anticodon loop [38].

Pseudouridine (Ψ), was discovered over 60 years ago [39,40] and was originally called the “fifth nucleotide”. This is the most abundant modified ribonucleotide in total cellular RNA [[39], [40], [41]] and is formed through isomerization of uridine, generating a C—C glycosidic bond instead of the usual C—N, with the now available N1—H acting as an additional hydrogen bond donor on the Hoogsteen edge. This results in a restricted nucleobase conformation and backbone mobility, and greater stability of RNA duplexes where Ψ occurs [42,43].

### 1.3 Post-transcriptional RNA modifications in mitochondria: overview

Mitochondrial gene expression has some unique features compared to cytosolic RNA metabolism [44]. The mitochondrial genome encodes the reduced set of only 22 mt-tRNAs to decode 60 different codons. The necessary decoding flexibility is presumably achieved through a range of complex tRNA modifications and will be discussed further in detail in this review (Section 3). Similarly, the high protein:RNA ratio observed in the mitochondrial ribosome compared to all other ribosomes, makes correct folding and high stability of the mt-rRNA particularly critical to ensure accurate mitoribosome biogenesis and function. Similarly to bacteria and to some modifications in eukaryotic cytoplasmic rRNAs, mitochondrial rRNA modifications depend on site-specific enzymes that are not guided by small nucleolar (sno) RNAs [45]. In mt-rRNAs, the modifications cluster in functionally important regions, such as the decoding centre, mt-tRNA binding sites and the peptidyltransferase center [46]. This leads to the suggestion that these RNA modifications may play a role in translational control of mitochondrial gene expression. In this review we will also discuss the current knowledge of mt-rRNA modifications and their role in mitochondrial function (Section 2).

The precise function of the majority of the modifications is far from understood. It has however been shown that important cellular processes are dependent on the presence of modified cytoplasmic nucleotides and lack of modification results in human disease (*e.g.* intellectual disability, cancer, type-2 diabetes and obesity [47]). Similarly, the absence of mtRNA modifications, due to mutations at or near mtRNA sites or in mtRNA modifying enzymes, has also been associated with human mitochondrial pathologies [[48], [49], [50], [51], [52]]. However, the role of mtRNA modifications in human disease is not described in this review as this topic is comprehensively covered in other recent reviews [30,[53], [54], [55], [56], [57]].

## 2 Mitochondrial rRNA modifications

Mitoribosomes are composed of two types of components: (i) a core rRNA molecule, 12S for the small 28S subunit (mtSSU) and 16S for the large 39S subunit (mtLSU), and (ii) peripheral components, mostly proteins. These peripheral components extensively decorate the solvent accessible surface of these core rRNA molecules, apart from the regions involved in the interaction with mt-tRNAs, the mt-mRNA molecule being translated, and the interface between the mitoribosomal subunits. Additionally, the mtLSU has a peripheral mtDNA-encoded RNA component, which is structurally analogous to the 5S rRNA, and can either be mt-tRNA^Val^ in *Homo sapiens* and *Mus musculus*, or mt-tRNA^Phe^ in *Sus scrofa* and *Bos taurus* [[58], [59], [60]]. Compared to their bacterial ancestors, mitoribosomes present a higher protein:RNA ratio as a result of the reductive evolution of the mitochondrial genome, including several deletions in mt-rRNAs and structural replacement of the eliminated segments by residues of mitoribosomal proteins [61,62]. Compared to the cytosolic homologues, nuclear-encoded mitoribosomal proteins present unique extensions that structurally make up for the lost RNA portions.

Several factors are required to facilitate the biogenesis of functional ribosomal subunits. This includes the repertoire of enzymes that are responsible for epitranscriptional modifications and maturation of the core RNA molecules. In contrast to Archaea and the cytosol of eukaryotic organisms [43,63,64], pseudouridylation and 2′-*O*-methylation of bacterial RNAs and eukaryotic mtRNAs are performed in a small nucleolar ribonucleoprotein (snoRNP)-independent manner, resorting to enzymes that recognise the target nucleotide *via* its structural context. Adding to this, the selectivity and relatively limited extension of the mitochondrial proteome also accounts for the reduced number of modifications in mt-rRNAs. Still, the modifications that are preserved in mitoribosomes seem to be sufficient to produce a functioning macromolecular complex capable of translation [36].

Among the diverse repertoire of RNA modifications, only three types of modifications have been observed in mt-rRNAs: nucleobase methylation, 2′-*O*-methylation and pseudouridylation. Furthermore, the number of modified nucleotides in the mt-rRNAs is considerably reduced relatively to ribosomes of other compartments and organisms, but those remaining present a high degree of conservation, which indicates that although sparse, these modifications retain structural and/or mechanistic relevance for translation. Disruption of the pathways involved in formation of these modifications is therefore expected to have a functional impact in mitochondrial and cell homeostasis [45,65].

Since the majority of mammalian mt-rRNA modifications were first mapped in cultured BHK-21 hamster cells [[66], [67], [68], [69], [70], [71]] the field of mitochondrial epitranscriptomics has moved forward, both expanding our knowledge on previously identified modifications and uncovering new modifications for future research. To date, the enzymes responsible for eight of the ten mt-rRNA modifications have been identified. Most of the mt-rRNA modifications detailed below have been confirmed to be present in human mitoribosomes. Still, while the body of knowledge regarding the cellular impact of these modifications, or lack of thereof, has been building, their mechanistic role is still poorly understood.

### 2.1 Modifications of the large mitoribosomal subunit

#### 2.1.1 m^1^A947

The m^1^A947 (human mtDNA position: m.2617A) modification is the only case of nucleobase methylation in 16S mt-rRNA, which otherwise is enriched in ribose 2′-*O*-methylation. It appears specific to mitochondria as the corresponding residues in other types of ribosomes are not modified [64]. This modification was first identified in human as unexpected mismatches between data from RNA-Seq experiments and the corresponding mtDNA reference sequence, and were thus named RNA-DNA differences (RDDs). These events would appear as if prevalent A-to-U (>30%) and A-to-G (~15%) mtRNA editing events, already present in the polycistron containing 16S mt-rRNA, although at lower levels, took place; however, this artefact arises from the erroneous nucleotide incorporation by the reverse transcriptase when trying to base-pair an adenine nucleotide with an unusual Watson-Crick edge topology. The A-to-U conversion was observed in six human cell types and is conserved across representative primates, while the primal ancestral allele presents a T in this position. Since presence of an A at this position is highly correlated with observation of RDD across species [72], and no RDD was detected in species where the mtDNA sequence bears a T allele, it was hypothesised that the A-to-U RNA editing/modification event serves to recapitulate the product of the primate ancestral T allele [73]. In a genome-wide association study, *TRMT61B* was found associated with this RDD detected in 16S mt-rRNA [74]. Initially, TRMT61B was characterised as the enzyme performing SAM-dependent nucleobase N^1^-methylation of A58 in mt-tRNAs, which confers a stabilising positive charge to the elbow of these RNAs. Since the methyl group of m^1^A is deposited in the Watson-Crick face of the nucleobase, it disrupts the canonical base pairing with U; although m^1^A58 is still able to base pair m^5^U54 of the T-loop *via* a reverse Hoogsteen interaction [75]. Additional involvement of TRMT61B in the generation of m^1^A947 was confirmed by reverse transcription-primer extension and LC-MS of human 16S mt-rRNA from cells where TRMT61B or TRMT10C (also involved in the synthesis of m^1^A in mt-tRNAs, see Section 3) were knocked down by siRNA, and in *in vitro* reconstitution experiments. The fact that TRMT61B was able to recognise and act upon naked RNA further corroborates the hypothesis that m^1^A947 is generated in 16S mt-rRNA during the early steps of mitoribosome assembly. Additionally, dependency between m^1^A947 and TRMT61B was also observed when the RDD associated to this position was not detected by RNA-seq upon knock-down of this enzyme [72].

**Fig. 1:**
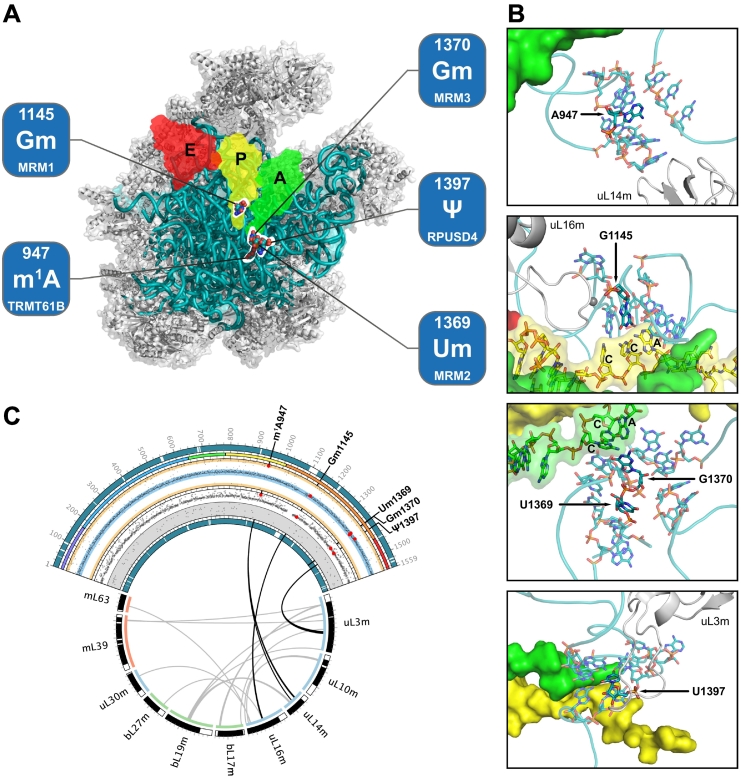
Modifications of the mtLSU rRNA. (A) View of the mtLSU from the interface between mitoribosomal subunits. 16S mt-rRNA modifications are localised in the three-dimensional structure of the mtLSU, and the dedicated enzymes appointed. Putative localisation of tRNAs (green, yellow, red) is presented from structural alignment of a bacterial ribosome loaded with these RNAs (PDB ID: 5JTE [209]). 16S mt-rRNA is presented as a teal ribbon and MRPLs as grey cartoons. (B) Local environment of each mt-rRNA modification (identified in bold and pointed by an arrow). Relevant MRPLs are also identified. Structurally aligned RNAs are presented as surfaces with the same colour coding as in (A). (C) Representation of 16S mt-rRNA (teal), its modifications (bold) and their association to relevant MRPLs. From the outside to the inside: coverage of the 16S mt-rRNA, rRNA domains, nucleotide ribose pucker represented by its pseudorotation angle (yellow areas: C2′-*endo* and C3′-*exo*; blue areas: C3′-*endo* and C2′-*exo*), nucleobase conformation (white area: *anti*; grey area: *syn*), coverage of the presented molecules is reported as coloured or black arcs, phylogenetic origin of the presented MRPLs (blue: universal; green: bacterial; red: mitochondrial), putative interactions (black: RNA-MRPL contacts, thresholded to 10 Å; grey: MRPL-MRPL contacts, thresholded to ~3 Å). Data points relative to the modified residues are presented as larger red dots. Ticks span 25 nucleotides in the 16S mt-rRNA arc, and 25 amino acids in MRPL arcs. Human mitoribosomal structures and coordinates were obtained from PDB ID: 3J9M [61].

In the mammalian mtLSU, A947 is located in the structurally conserved helix 71 of domain IV, which bears some degree of sequence similarity (UAAAU) with the consensus found in the T-loop of the six mt-tRNAs modified by TRMT61B (YMRAW) [76] (Fig. 1). This residue is positioned in such a way that interactions with the negatively charged phosphate backbone of helix 64 are enabled, and further electrostatically stabilised by the positive charge added by the N^1^-methylation. Furthermore, helix 71 is located near an intersubunit bridge (B3) and contacts helix 92 of domain V (which is in turn stabilised by ribose 2′-*O*-methylation of U2552 in *Escherichia coli* [77]). Phylogenetic studies revealed that this position can be occupied by an unmodified U (human cytoplasmic ribosomes and 10% of vertebrate mitoribosomes), unmodified G (in 95% of bacterial ribosomes) or m^1^A (90% of vertebrate mitoribosomes) [72]. *In silico* modelling of A in the corresponding position of 23S rRNA in the *Escherichia coli* ribosome structure (G1954) reveals a destabilisation in the local environment of helix 71, by abolishing a stabilising hydrogen bond with helix 64, while this interaction is preserved when U or G occupy the position [73]. Corroborating evidence was also found when the target residue was mutated *in vivo*, with the A1954 strain presenting slower growth compared to U1954 and G1954 due to impaired protein synthesis (decreased synthesis rate and reduced total protein production). In the case of the bacterial ribosome, with G1945, the exocyclic amino group of guanine (N2) may act as a proton donor, thus having the potential to emulate the stabilising interactions established by m^1^A947 in the human mitoribosome. Despite the difference in the size of the nucleobase, U1945 may also contribute to the stabilisation of its microenvironment by a different mechanism that involves an indirect interaction with the rRNA backbone of helix 64 *via* water molecules [72].

TRMT61B is an example of divergent evolution after gene duplication, with acquisition of a novel target. While TRMT61B is responsible for the N^1^-methylation of A58 in mt-tRNAs and A947 in 16S mt-rRNA, the bacterial TrmI and yeast Trm61p orthologues, and the vertebrate paralogue TRMT61A are only responsible for the biosynthesis of m^1^A58 in cytosolic tRNAs. These proteins act as tetramers: TrmI and TRMT61B form homotetramers in bacteria and mitochondria, respectively, and heterotetramers in the cytosol of eukaryotes, Trm61p/Trm6p in yeast and TRMT61A/TRMT6 in vertebrates. Additionally, eukaryotic TRMT61B displays higher similarity to the bacterial TrmI than its cytosolic paralogue TRMT61A [64,75,78,79].

#### 2.1.2 Gm1145

Modification of the G1145 (human mtDNA position: m.2815G) nucleotide is highly conserved across ribosomes of different species. It is localised in the P-loop of the mt-rRNA (Fig. 1), which is a structural and functional part of the peptidyl transferase centre (PTC). G1145 is involved in the interaction with the tRNA carrying the nascent polypeptide. All these features strengthen the hypothesis of this modified nucleotide being one of the crucial residues for translation by maintaining the active conformation of the residues contacting the tRNAs contained within the ribosome [64].

In *Escherichia coli*, the ribose 2′-hydroxyl of G2251 of 23S rRNA (equivalent to human G1145 from 16S mt-rRNA) is methylated by RlmB/YjfH [80]. This non-essential protein forms a dimer in solution and is composed of a N-terminal domain that is likely to be involved in 23S rRNA recognition due to its similarity to bL12 and uL30, and a C-terminal catalytic SpoU-like guanosine methyltransferase domain, both connected by a flexible linker [81].

Deletion of RlmB in bacteria did not produce any appreciable defects in the stability of ribosomal subunits, their assembly nor any appreciable growth delay [80]. Still, RlmB may adopt a more relevant role under certain stress conditions, in a similar fashion to other methyltransferases that confer antibiotic resistance to the ribosome by modifying its RNA component and preventing its inhibition [81].

In the genome of *Saccharomyces cerevisiae*, *pet56*, the yeast orthologue of *rlmB*, is adjacent to the functionally unrelated gene *his3*, which encodes an enzyme involved in the biosynthesis of histidine. These genes are bidirectionally transcribed from an A/T-rich intergenic region bearing the promoters of both genes in close proximity. In contrast to the bacterial system, perturbation of the promoter of *pet56* produces the respiratory-deficient *petite* phenotype [65,82], revealing the relevance of this gene for mitochondrial function. Characterisation of the N-terminus of Pet56p/Mrm1p, which is enriched in hydrophobic and basic amino acids while devoid of acidic residues, thus physicochemically resembling other mitochondrial targeting sequences, further corroborated that not only is this protein crucial for mitochondrial homeostasis, but also that it is likely to be imported into this organelle [65,83]. Disruption of *pet56* expression leads to a markedly slow growth in non-fermentable carbon sources and decreased abundance of proteins of the mitoribosomal large subunit. Altogether, this body of data indicates that Pet56p/Mrm1p acts upon mitochondrial function by impinging on the translation machinery of this organelle. While the stability of the yeast 21S mt-rRNA was unaffected by deletion of *pet56*, methylation of G2270 (equivalent to G1145 in the human 16S mt-rRNA) was absent when inspected by reverse transcription-primer extension. Methylation of *in vitro* transcribed rRNA by recombinant Pet56p/Mrm1p in a SAM-dependent manner provided strong evidence that this protein is involved in the generation of Gm2270. Modification of naked rRNA by recombinant Pet56p/Mrm1p is consistent with this protein acting during the early stages of mitoribosome biogenesis [65]. Analysis of the mitoribosomal integrity in strains where *pet56* is disrupted led to the observation of a considerable decrease in the amount of mature mtLSU and an accumulation of lower density species, likely to be partially assembled mtLSU or disassembly/dissociation products, containing uL3m and uL23m, but not bL27m [45,65].

The human orthologue of Pet56p/Mrm1p, MRM1, has been shown to introduce a methyl group in the ribose 2′-hydroxyl of G1145 by the inspection of DNAzyme fragmentation products and reverse transcription-primer extension. This protein was found to co-sediment and remain associated with mitochondrial nucleoids in density gradients, while presenting a weak interaction with mitoribosomes [84]. However, further functional characterisation is lacking for this mitochondrial methyltransferase.

Information on the microenvironment of residue G1145 has been obtained by chemical probing [85] and structural insights [60,61], revealing its placement within the peptidyl transferase centre in the P-loop, with G1145 and its neighbouring nucleotides being involved in contacting the 3′-terminual extension of the P-site tRNA, namely its post-transcriptionally added CCA extension.

#### 2.1.3 Um1369

Um1369 (human mtDNA position: m.3039 T) is located in the A-loop of the mtLSU domain V, near the entrance to the nascent peptide tunnel, in close proximity to where the 3′ CCA terminal of the incoming tRNA would lie within the mitoribosome (Fig. 1). The nucleobase of this residue is pointing away from the tRNA, establishing interactions with other residues, while the ribose 2′-hydroxyl is more proximal to the tRNA, possibly having a role in stabilising the structure of the loop.

The bacterial enzyme responsible for the 2′-*O*-methylation of U2552 in 23S rRNA from *Escherichia coli* (equivalent to U1369 of the human 16S mt-rRNA), FtsJ/RlmE, has been extensively studied and its ablation has been shown to lead to severe growth defects and a thermosensitive phenotype. The *ftsJ/rlmE* open reading frame is part of an operon in the *E. coli* genome together with the protease *ftsH* and its transcription is highly upregulated upon heat shock *via* the σ^32^ transcription factor [86]. Presence of a SAM-binding motif containing conserved residues, identification of SAM by mass spectrometry in purified protein, and adoption of a spatial fold similar to that of several methyltransferases (seven-stranded β sheet flanked by five α helices), with a putative substrate binding groove composed of conserved positively charged residues strongly suggested the catalytic activity of FtsJ/RlmE. In search for the substrate of this enzyme, DNA, mRNA, bacterial 16S rRNA and 23S rRNA were tested *in vitro* for methyl deposition, from which only the latter was able to be modified but only when in the context of 50S LSU ribosomal particles isolated from the deletion strain. Analysis of the equilibrium profile of ribosomal subunits in sucrose gradients showed a defect in the assembly of these RNPs, with accumulation of 30S and also 45S particles dependent on the concentration of Mg^2+^, and a decrease in the quantity of 70S and polysomes [77,[86], [87], [88]]. The 45S particle observed under stringent Mg^2+^ concentrations lacks the ribosomal protein bL36 and other downstream ribosomal proteins. However, methylation of the 2′-*O*-ribose of 23S rRNA U2552 by FtsJ/RlmE permits the progression of the late stages of maturation of the 45S intermediate to 50S by allowing the establishment of an interdomain association between helixes H92 (where the modified residue is located) and H71, and incorporation of bL36 into the assembly [77]. An additional phenotype of RlmE-null bacterial mutants is a decrease in translational ±1 frameshifting and readthrough of stop codons, which points towards an increase in translation accuracy at the expense of the efficiency of this process when U2552 is not modified [89]. A complementation screen performed on ∆*rlmE* strains identified two GTPases, EngA and ObgE, whose overexpression could specifically supress the phenotype observed in the null mutants (*i.e.* restoration of growth rate, mitigation of thermosensitivity, normalisation of the levels of ribosomal subunits, and restoration of 70S ribosome and polysomes). Incubation of wild-type derived 50S subunits with RlmE in an *in vitro* methylation assay, resulted in low levels of methyl incorporation, which suggests that the ribose 2′-hydroxyl of U2552 is typically methylated to near completion. Nonetheless, LSU from ∆*rlmE* and the null strains complemented with EngA and ObgE (which presents high similarity to human mitochondrial GTPBP5 and GTPBP10 [90]) showed high incorporation of labelled methyl, indicating that despite the phenotype suppression, the 50S (*i.e.* mature/assembled LSU) of the complemented strains does not harbour a modified U2552 residue [88].

Mutation of U2552 to A, C or G in 23S rRNA from *Escherichia coli* leads to no appreciable growth defect, which points to the little relevance of the nucleobase of this residue to its function. However, since hypomodification of this position leads to severe phenotypic changes, it can be inferred that the mechanistic relevance of U2552 (U1369 in human 16S rRNA) lies on the methylated ribose 2′-hydroxyl [91].

Inspection of the *S. cerevisiae* proteome identified three paralogues of the bacterial FtsJ methyltransferase: Spb1p, Trm7p and Yg1136c. From these, Spb1p, an ssRNA-binding protein that is involved in the coordination between mRNA translation and decay, was shown to be a nucleolar S-adenosylmethionine (SAM)-binding protein required for the biogenesis of the cytosolic LSU 25S rRNA, and Trm7p was reported to methylate the 2′-*O*-ribose of the anti-codon loop residues 32 and 34 of cytosolic tRNAs. Yg1136c was reported to be a nuclear-encoded mitochondrial protein, with *in vivo* and *in vitro* 2′-*O*-methyltransferase activity for U2791 in 21S mt-rRNA, and thus was renamed as mitochondrial rRNA methyltransferase 2 (Mrm2p). Similarly, to the bacterial deletion strain, the *mrm2*Δ yeast strain shows a thermosensitive growth phenotype, exacerbated in media containing a non-fermentable carbon source. Furthermore, continuous growth in glucose-containing medium leads to the appearance of *petite*-like colonies characterised by mtDNA depletion, which is generally a typical presentation of dysfunctional mitochondrial translation [92]. Mrm2p was found to co-fractionate with the mtLSU 21S mt-rRNA in sucrose gradients and 2′-*O*-methylated U2791 was absent in the *mrm2*Δ strain when RNA was analysed by reverse transcription-primer extension, which corroborated the previous findings and identified this protein as a regulator of mitochondrial translation, explaining the generalised impact on mitochondrial function upon its depletion. Curiously, as in bacteria, methylation of U2791 was able to be recapitulated *in vitro* when mtLSU particles isolated from *mrm2*Δ were incubated with affinity-purified Mrm2p in the presence of SAM, but not when deproteinised rRNA was used, which indicates that this methyltransferase acts on assembled mtLSU and/or late stage assembly intermediates [93].

With this background, the human orthologue of FtsJ/RlmE and MRM2p was identified, while initially named FTSJ2, its designation later changed to MRM2. Analysis of the human protein revealed a putative mitochondrial targeting sequence (MTS) as well as a FtsJ-like uridyl-2′-*O*-methyltransferase domain. Reverse transcription-primer extension and the use of DNAzymes demonstrated that this protein is responsible for the modification of U1369 of the human 16S mt-rRNA [84,94,95]. Immunopurification of MRM2 revealed its interaction with the mitoribosome, with both mtLSU and mtSSU proteins being co-purified. In the reciprocal immunopurification experiment, MRM2 was pulled-down by mL62 and mL27. However, since these two mitoribosomal proteins are also able to pull-down the 55S mitochondrial monosome, it is possible that MRM2 binds to the mtLSU, the mitochondrial monosome or both. Inspection of the distribution of MRM2 in sucrose density gradients corroborates this hypothesis as this protein is seen to co-sediment with the mtLSU and denser assemblies. Upon silencing of MRM2 in cultured human cells, the steady state levels of mtLSU (by proxy of uL3m and bL12m) was found to be decreased with no apparent changes regarding the mtSSU. Also, mitochondrial protein synthesis and respiration were considerably decreased, as well as the growth of MRM2-depleted cells, especially under OxPhos eliciting conditions (by using glucose-free medium containing galactose as the sole carbon source) [94].

Given these findings, 2′-*O*-methylation of U1369 may be relevant in quality control mechanisms of mitochondrial translation, not only given the strategic location of this modification at the entrance of the nascent peptide tunnel of the mitoribosome, possibly exerting control over the peptidyl transferase centre and/or the incoming mt-tRNAs, but also by possibly constituting a checkpoint or licensing step in the assembly of these macromolecular complexes.

#### 2.1.4 Gm1370

Along with Um1369, Gm1370 (human mtDNA position: m.3040G) is also located in the A-loop of the mtLSU, although closer to the entrance of the peptide exit channel (Fig. 1). The nucleobase of this nucleotide adopts a conformation that allows its interaction with the 3′ terminal CCA of mt-tRNAs loaded in the A-site of the mitoribosome. As such, and given that tRNAs lacking CCA are unable to participate in translation, Gm1370 may exert a quality control over the input tRNAs during translation, by allowing only mature tRNAs to be stably situated in the A-site or the active mitoribosome, and/or by using the 3′ terminus of the mt-tRNA to position the amino acid residue in place for the peptidyl transferase reaction.

The enzyme responsible for 2′-*O*-methylation of G1370 is the methyltransferase MRM3 (also known as RMTL1 or RNMTL1), which was identified in a proteomics characterisation of cross-linked mitochondrial nucleoids [96]. The observation of evolutionary co-occurrence of 2′-*O*-methylated G1370 and G1369 and orthologues of MRM3 or MRM2, respectively, strengthened the hypothesis of these proteins being implicated in mt-rRNA modification, at the same time suggesting their targets [94]. MRM3 has a similar domain architecture relative to MRM1, with a N-terminal 2′-*O*-ribose binding domain and a C-terminal SpoU-type guanosine methyltransferase domain. Furthermore, the N-terminal region of this protein has been assigned with a high probability of containing a mitochondrial targeting sequence (MTS) [94,95]. Separation of mitochondrial constituents by ultracentrifugation in density gradients that maintain nucleoids intact revealed that MRM3 co-sediments with these structures and with mtLSU; the MRM3:mtLSU interaction persists even in the presence of EDTA, which is known to cause subunit dissociation by chelating structural Mg^2+^ from the ribosome. Additional characterisation of MRM3 by co-immunopurification identified several constituents of the mitoribosome (mostly from the mtLSU), as well as other mtRNA-related proteins as interactors of this methyltransferase, including the RNA chaperones (*e.g.* p32/C1QBP, LRPPRC or GRSF1), mitoribosome assembly factors MTERF3, DDX28, and the 16S mt-rRNA ψ synthase RPUSD4 (see Section 2.1.5) [95].

Similarly to MRM2, the knock-down of MRM3 leads to decreased mitochondrial translation with consequent OxPhos deficiency, and impaired mitochondrial respiration and growth in galactose-containing medium. When immunopurified, mainly mtLSU components were detected as interacting with MRM3, with mS27 not being able to pull-down this methyltransferase; this not only presents the specificity of MRM3 towards binding the mtLSU, but also demonstrates that it interacts with this subunit at a stage where it is not in the context of the 55S mitochondrial monosome. Investigation of the profile of mitoribosomal subunits in sucrose density gradients upon MRM3 knock-down revealed a shift of mtLSU (by proxy of uL3m and bL12m) towards less dense fractions, potentially corresponding to an assembly intermediate that is accumulated when the maturation of this subunit is not allowed to progress due to the lack of MRM3 [94].

Mutation of G2553 in *E. coli* 23S rRNA (equivalent to G1370 of the human 16S mt-rRNA) to A, U or C leads to growth defects accompanied by loss of interactions with tRNA and elimination of peptidyl transferase activity. This finding not only points towards the importance of residue 2553 being a guanosine for the mechanism of translation, but also specifies the nucleobase of this residue as having a major functional relevance for translation, by mediating the interaction between the ribosome and the 3′ CCA terminus of A-site tRNAs [91]. Notwithstanding, the role of the 2′-*O*-methylation cannot be inferred from bacterial studies for G2553 nor from the studies of the corresponding residue in the yeast mtLSU, as these organisms lack methylation in these positions [64,94]. Although guanosines in the equivalent position in the yeast (25S rRNA G2922) and human (28S rRNA G4499) cytosolic ribosomes are also a target for 2′-*O*-methylation, this modification is deposited *via* snRNA-guided mechanisms in the former, while the entities responsible for it are still unknown for the latter [64].

#### 2.1.5 Ψ1397

The 16S mt-rRNA contains a single known pseudouridine - Ψ1397 (human mtDNA position: m.3067T) [97]. This site is located in domain V of mtLSU, near the core of the peptidyl transferase centre, in a less solvent-accessible region of the 16S mt-rRNA, but still in close proximity with Um1369 and Gm1370, contributing to the second shell of interactors of these residues (Fig. 1). Although the bacterial pseudouridine synthases are well characterised, the identification of the mitochondrial counterparts and their respective RNA targets has remained elusive until a CRISPR/Cas9 screen for genes essential for OxPhos [98]. This study reported the implication of three putative pseudouridine synthases, TRUB2, RPUSD3 and RPUSD4, in mitoribosome biogenesis. The same study suggested the participation of these three pseudouridine synthases, together with other RNA-binding proteins (FASTKD2, NGRN, WBSCR16), in a functional module that is critical for the stability of 16S mt-rRNA and, consequently, mitochondrial translation [98]. Silencing of RPUSD4 revealed defects in steady state levels of OxPhos complexes and decreased mitochondrial respiratory capacity, with unaffected mt-mRNA levels but with a significant and specific reduction in the quantity of 16S mr-rRNA and assembled mtLSU, further pointing towards a defect in mitochondrial translation. Cross-link immunopurification (HITS-CLIP) identified RNA binding sites for this pseudouridine synthase, one of which in the vicinity of position 1397 in the *MT-RNR2* transcript, which was identified to be pseudouridylated [99]. Single-nucleotide resolution Psi-Seq was able to further narrow down the functional target of RPUSD4 to this nucleotide by observing polymerase skipping events in the reverse transcription products from CMC-modified RNA from control cells, corresponding to the presence of Ψ1397. The significant decrease of the number of skipped reads in cells where RPUSD4 was silenced identified this protein as the one responsible for the generation of Ψ1397 in 16S mt-rRNA [100]. Curiously, even though TRUB2 and RPUSD3 were not found to modify mt-rRNA, but rather mt-mRNAs (see below), a selective 16S mt-rRNA/mtLSU depletion phenotype similar to that of RPUSD4 knock-down was observed when these two mitochondrial pseudouridine synthases were silenced. Co-localisation studies with FASTKD2, a *bona fide* MRG protein, identified RPUSD4 in these intra-mitochondrial structures, further corroborating the hypothesis that these intra-mitochondrial structures serve as hubs for the maturation, assembly and biogenesis of mitochondrial ribosomes [99].

### 2.2 Modifications of the small mitoribosomal subunit

#### 2.2.1 m^5^C841

**Fig. 2:**
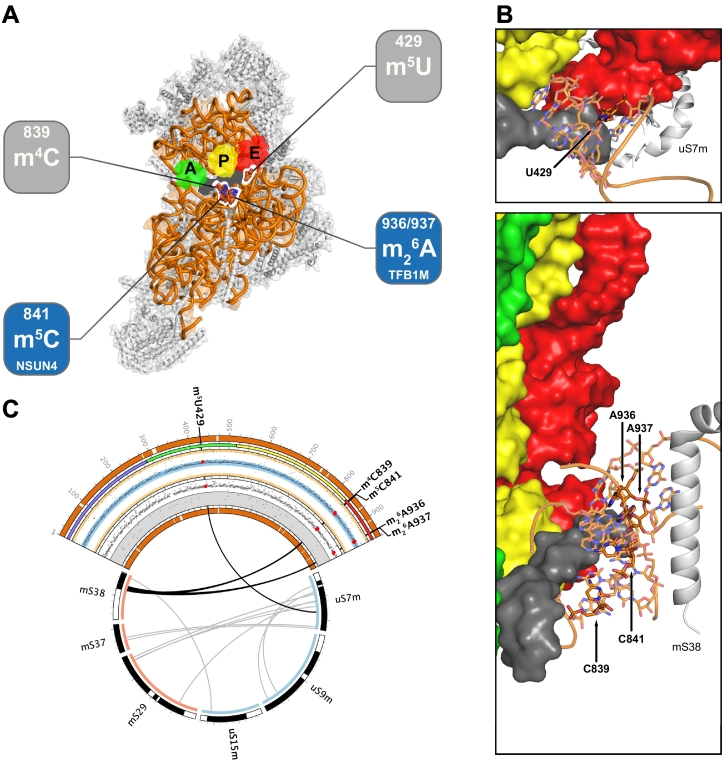
Modifications of the mtSSU rRNA. (A) View of the mtSSU from the interface between mitoribosomal subunits. 12S mt-rRNA modifications are localised in the three-dimensional structure of the mtSSU, and the dedicated enzymes appointed. Putative localisation of tRNAs (green, yellow, red) and mRNA (dark grey) are presented from structural alignment of a bacterial ribosome loaded with these RNAs (PDB ID: 5JTE [209]). 12S mt-rRNA is presented as an orange ribbon and MRPSs as grey cartoons. (B) Local environment of each mt-rRNA modification (identified in bold and pointed by an arrow). Relevant MRPSs are also identified. Structurally aligned RNAs are presented as surfaces with the same colour coding as in (A). (C) Representation of 12S mt-rRNA (orange), its modifications (bold) and their association to relevant MRPSs. From the outside to the inside: coverage of the 12S mt-rRNA, rRNA domains, nucleotide ribose pucker represented by its pseudorotation angle (yellow areas: C2′-*endo* and C3′-*exo*; blue areas: C3′-*endo* and C2′-*exo*), nucleobase conformation (white area: *anti*; grey area: *syn*), coverage of the presented molecules is reported as coloured or black arcs, phylogenetic origin of the presented MRPSs (blue: universal; red: mitochondrial), putative interactions (black: RNA-MRPS contacts, thresholded to 10 Å; grey: MRPS-MRPS contacts, thresholded to ~3 Å). Data points relative to the modified residues are presented as larger red dots. Ticks span 25 nucleotides in the 12S mt-rRNA arc, and 25 amino acids in MRPS arcs. Human mitoribosomal structures and coordinates were obtained from PDB ID: 3J9M [61].

Pioneering studies aimed at detecting modified residues in mammalian mitochondrial rRNAs led to the identification of a single 5-methylcytosine residue in the mtSSU (human mtDNA position: m.1488C) (Fig. 2) [70]. This type of RNA modification is generally deposited by the members of the NOP2/Sun RNA methyltransferase family, which is transversal to all kingdoms of life. Mammalian genomes code at least seven conserved members of this family, which share a seven β-strand fold and use SAM as the methyl donor for the generation of 5-methylcytosine in cytosolic and mitochondrial non-coding RNAs, including t- and rRNAs [52].

The bacterial methyltransferase RsmF/YebU, modifies the corresponding residue (C1404 in *E. coli* numbering) in *Thermus thermophilus* 16S SSU rRNA, alongside two other cytosines (C1400 and C1407). However, the *E. coli* RsmF/YebU is only responsible for the formation of a single modification in the SSU rRNA, m^5^C1407. These three residues are located in helix 44, near the highly conserved decoding centre of the SSU. Lack of RsmF in *T. thermophilus* leads to thermosensitive growth phenotype. *In vitro* methylation studies revealed that RsmF is able to modify ~35% of naked 16S rRNA at C1404, while this position is found to be completely modified when the same rRNA is presented in the context of 30S SSU, indicating the involvement of this protein at later stages of ribosome biogenesis. Interestingly, C1407 is only able to be methylated by RsmF in the context of 30S particles. In order to perform its catalytic activity on C5 of the nucleobase of cytosines, RsmF requires the base to be unpaired. Similarly to the equivalent residue in the mammalian mitoribosome, C1404 is engaged in a Watson-Crick pairing in helix 44 and buried within the 30S subunit, thus hindering direct access to C5 of this residue and possibly implying the necessity for some degree of structural rearrangement involved in the synthesis of m^5^C1404 [101].

The eukaryotic orthologue NSUN4 has been identified in mouse as the methyltransferase responsible for the generation of m^5^C841; confirmation in a human system is missing. Knock-out of the *NSUN4* coding gene is embryonic lethal in mice, while conditional knock-out in the heart leads to increased mitochondrial mass, mtDNA copy number and levels of mitochondrial *de novo* transcription, with decreased mitochondrial translation and accumulation of mtSSU and mtLSU [102].

NSUN4 interacts stoichiometrically with MTERF4, a member of a family of proteins involved in the regulation of mtDNA gene expression, to form a heterodimer that is crucial for the quality control of the last stages of mitoribosome biogenesis, namely the association between mature mtSSU and mtLSU to form translationally functional mitochondrial monosomes. Absence of MTERF4 leads to a similar phenotype to that of NSUN4 knock-out [102], with increased levels of mitochondrial transcripts and a concomitant decrease in mitochondrial translation due to reduced association between mitoribosomal subunits and thus formation of functional mitoribosomes, even though the quantity of mtSSU and mtLSU are increased and NSUN4 is still able to modify its target residue. Furthermore, unlike its prokaryotic counterpart, NSUN4 lacks RNA-binding domains, relying on MTERF4, which binds primarily to 16S mt-rRNA, to be recruited to the mitoribosome, as evidenced by the decreased quantity of NSUN4 associated with the mtLSU upon MTERF4 knock-down [103]. The low dissociation constant found for the MTERF4:NSUN4 complex suggests that both proteins exist in the mitochondrial matrix as a heterodimer. In this form, the *in vitro* methyltransferase activity of NSUN4 is greatly enhanced, while the monomeric enzyme presents significantly lower, intrinsic activity; although the monomeric NSUN4 is still able to deposit methyl groups on the substrate, it does so with lower specificity. In terms of RNA binding, a strongly positively charged concave patch on MTERF4 is thought to perform the initial docking of the complex to mt-rRNA, and this is then threaded into the catalytic site of NSUN4 through two grooves along the surface of the complex, one formed in the interface of the two proteins, and another formed by NSUN4 alone, leading to the proximity of the SAM binding pocket [104,105].

#### 2.2.2 m_2_^6^A936/m_2_^6^A937

The adjacent m_2_^6^A936/m_2_^6^A937 (human mtDNA position: m.1583A and m.1584A, respectively) residues are universally conserved, and are located in the loop of a conserved hairpin in helix 45, at the 3′ terminal of 12S mt-rRNA (Fig. 2). Their SAM-dependent N^6^-dimethylation is transversal from bacterial to eukaryotic cytosolic and mitochondrial ribosomes, and TFB1M has been identified as the protein responsible for their synthesis [7,9,64,106].

TFB1M (mtTFB1) is one of the two homologues of the bacterial methyltransferase KsgA/RsmA. Along with its paralogue, TFB2M (mtTFB2), these two proteins have been found to be critical in the regulation of the expression of the mitochondrial genome at different levels. TFB2M interplays with TFAM and the mitochondrial RNA polymerase (POLRMT) during mtDNA transcription, whereas TFB1M controls mitoribosome biogenesis *via* modification of 12S rRNA [6,7,106,107]. Due to their control over key processes, the genes encoding TFB1M and TFB2M are under direct control of the mitochondrial biogenesis master regulators NRF-1 and NRF-2, as well as the PGC-1 family coactivators PGC-1α and PRC [106,108]. While retaining some methyltransferase activity, TFB2M regulates the expression of the mitochondrial genome by acting as a transcription factor and presents approximately 10 times more transcriptional activity compared to TFB1M [106]. However, upon TFB1M knock-down, mitochondrial translation is impaired, with no effect on mtDNA transcription or replication, and TFB1M overexpression does not result in increased mtDNA copy number, which is observed when TFB2M is overexpressed, further supporting the distinct roles of the two paralogues in mitochondrial gene regulation [106].

Loss of TFB1M is embryonic lethal in mice and conditional disruption of this gene completely abolishes the N^6^-dimethylation of A936 and A937, while also perturbing mitoribosome biogenesis and, consequently, mitochondrial translation [107]. *In vitro* experiments reveal that the association equilibrium of the bacterial large and small subunits is dependent on the methylation status of A1518/A1519 (equivalent to A936/A937 in human 12S mt-rRNA), with a preferential association of the large subunit to small subunits bearing the methylated residues [109]. Also, absence of the modified m_2_^6^Am_2_^6^A dinucleotide upon disruption of *ksgA*, indirectly renders the bacterial ribosome resistant to the aminoglycoside kasugamycin (ksg), which binds in the mRNA channel of the ribosomal small subunit and prevents P-site tRNA binding, thus inhibiting translation [110]. Comparison of ksg-sensitive and -resistant bacterial ribosomes revealed that the dimethylation of A1518/A1519 leads to an increased destabilisation of the hairpin due to base stacking of the modified nucleobases and consequent imposition of stereochemical strain to the tetraloop [[110], [111], [112]]. Inspection of the structure of translationally inactive 30Si particles with KsgA bound reveals that this enzyme binds to the SSU platform, interacting with rRNA helices 24 and 27 *via* its C-terminal, and to helix 45 *via* its catalytic N-terminal domain. This way, 30Si-bound KsgA blocks the interaction between helixes 44 and 45, which form the decoding centre in the mature ribosomal subunit [113]. Furthermore, the multiplicity of rRNA contacts established by KsgA can also explain the fact that this enzyme is able to modify 16S rRNA in the context of 30S subunits, and that naked 16S rRNA does not serve as a substrate for this enzyme [114].

Complementation of the *ksgA*-null bacterial strain with human TFB1M revealed to be efficient in functionally restoring the dinucleotide dimethylation and ksg sensitivity, unveiling the extent of the conservation of the involved epitranscriptomic mechanisms [9].

Although bacterial systems are often used to provide mechanistic clues to mitochondrial processes, the difference in complexity and context of these two systems cannot be overlooked, and while some parallels stand true, many differences exist. For instance, the ribosome-binding factor A (RbfA), necessary for the 5′ processing of the small subunit 17S rRNA precursor in bacteria, presents little sequence and functional similarity with its orthologue, RBFA, in the human mitochondrial system, where there are no intronic elements in mt-rRNAs. Still, mitochondrial RBFA plays an important role in mitoribosomal biogenesis by associating with the 3′ region of the 12S mt-rRNA, including the hairpin containing the m_2_^6^A936/m_2_^6^A937 residues. Knock-down of this factor leads to the hypomodification of residues A936 and A937, and while the non-modified mt-rRNA molecules are incorporated into mtSSU, they show decreased association with mtLSU, exerting a quality control over nascent mitoribosomes by preventing the formation of monosomes that are not translationally efficient [115]. Furthermore, the bacterial assembly factor Era binds to the 3′ end of the 16S rRNA, in a region downstream of the last hairpin of this rRNA, which contains the anti-Shine-Delgarno sequence, simultaneously occluding the binding site for bS1. This not only prevents the premature formation of the 70S monosome, but also prevents Shine-Dalgarno sequences of mRNAs from interacting with the complementary sequence of the 16S rRNA present in immature SSU [116]. Although mammalian mitoribosomes do not contain an anti-Shine-Dalgarno sequence, the orthologous ERAL1 still chaperones the 3′ terminal region of the corresponding 12S rRNA, however, with recruitment of the hairpin of helix 45 where the TFB1M target dinucleotide is located. Loss of ERAL1 in the human model leads to rapid and selective decay of 12S rRNA, and a consequent decrease in the amount of mature mtSSU [117]. The presence of overlapping binding sites for ERAL1 and TFB1M underlies a competitive mechanism in the assembly of the mtSSU, possibly with non-simultaneous and exclusive binding of different factors in different stages of this pathway. Also, the case of TFB1M further suggests the interplay between assembly factors and RNA-modifying enzymes, as well as the potential dual role of the latter in the biogenesis of functional ribosomal subunits capable of engaging in translation.

#### 2.2.3 m^5^U429 and m^4^C839

The function of 12S mt-rRNA m^5^U429 and m^4^C839 (human mtDNA position: m.1076 T and m.1486C, respectively) in mammalian mitochondria is still unclear. While the nucleotide equivalent to U429 is also modified in yeast and human cytosolic SSU, in both cases it is converted to pseudouridine, rather than m^5^U, and it is unmodified in the bacterial ribosome. In bacteria, C839 is uniquely modified by nucleobase and ribose methylation to m^4^Cm1402. The corresponding nucleotide in eukaryotic cytoribosomes is modified solely by snoRNA-guided 2′-*O*-methylation, contrasting with the nucleobase methylation-only observed in mitoribosomes [64]. Both modified residues are located proximal to the mRNA path (Fig. 2). U429 is located in the E site, possibly in the interface between the mRNA and the *E*-site mt-tRNA. On the other hand, C839 is located in the P-site and its nucleobase may interact with mRNA loaded in the mtSSU, potentially mediating an interaction as the exocyclic amino group that is targeted for methylation (N4) is pointing towards the mRNA path. In addition to the sequence proximity, C841 is also structurally nearby C839, also presenting its nucleobase to the mRNA path (Fig. 2). The enzymes responsible for these modifications in the 12S mt-rRNA are still to be identified.

## 3 Mitochondrial tRNA modifications

**Fig. 3:**
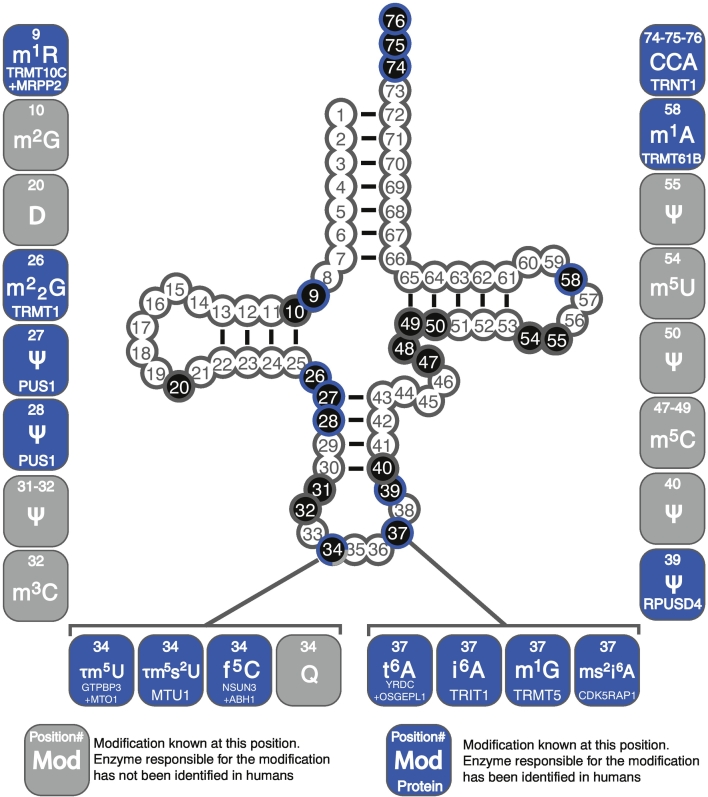
The maturation of mitochondrial tRNAs. Schematic of the cloverleaf secondary structure of a generic mitochondrial tRNA annotated with modifications so far identified in humans. Blue boxes: The modification and responsible enzyme(s) are known in human mt-tRNAs. Grey boxes: The modification is known in human mt-tRNAs, however the responsible enzyme is yet to be identified. Modifications shown: 1-methyladenosine (m^1^A), 1-methylguanosine (m^1^G), 1-methyladenosine or 1-methylguanosine (m^1^R), N2-methylguanosine (m^2^G), *N*2,*N*2-dimethylguanosine (m^2^_2_G), 3-methylcytosine (m^3^C), 5-methylcytosine (m^5^C), 5-formylcytosine (f^5^C), 5-methyluridine (m^5^U), 5-taurinomethyluridine (τm^5^U), 5-taurinomethyl-2-thiouridine (τm^5^s^2^U), *N*6-threonylcarbamoyladenosine (t^6^A), *N*6-isopentenyladenosine (i^6^A), 2-methylthio-*N*6-isopentenyladenosine (ms^2^i^6^A), Queuosine (Q), Pseudouridine (Ψ), Dihydrouridine (D), Cytidine-Cytidine-Adenosine trinucleotide (CCA).

The post-transcriptional maturation of tRNAs is undoubtedly the most varied and complex of all the RNA species known, with modifications occurring with the most frequency and the greatest chemical diversity. Here, mammalian mitochondrial tRNAs are no exception, with approximately 7% of all tRNA residues found to be modified with a wide range of modifications encompassing methylations, isomerisations, thiolations, formylations and ribosylations, among others, all performed by a multitude of nuclear encoded factors that require import into mitochondria (Fig. 3) [51]. Despite this, mitochondrially-encoded tRNAs are often considered oddities due to their sometimes dramatic divergence from well conserved features found in so-called ‘canonical tRNAs’ present in bacteria or eukaryotic cytosol. These peculiar structural properties of mt-tRNAs were immediately apparent following the first sequencing of the human mitochondrial genome [2], with the most striking found in mt-tRNA^Ser(AGY)^ which lacks an entire structural domain, the D-arm. Since then, metazoan mt-tRNAs showing further reduction, even lacking both the D- and the T-arms, have been uncovered [118]. Furthermore, the nucleotide bias present in the mitochondrial genome produces many mt-tRNAs that are A, U, and C-rich, while G-poor, resulting in fewer stem-stabilising G-C pairs compared to canonical tRNAs. As a consequence, the thermodynamic stability calculated *in silico* for the folding of mitochondrial tRNAs is found to be twice as low as that for canonical tRNAs [119]. Although further work on the post-transcriptional modifications of mt-tRNAs have found them to be characteristically abundant, they remain significantly lower than the number found in their cytosolic counterparts (11% for bacterial tRNAs, 17% for cytosolic eukaryotic tRNAs [120]), leading to the conclusion therefore that for a given mt-tRNA modification, its impact and necessity is greater than that for a modification in a canonical tRNA. For the purposes of this review, we will divide the post-transcriptional modifications made to mt-tRNAs into two groups: ‘core modifications’ that confer structural stability and correct folding, and ‘functional modifications’ that alter the manner in which the tRNA interacts with other factors in order to perform its role, although in reality such a clear distinction between structure and functional modifications is rarely present.

### 3.1 Core modifications

**Fig. 4:**
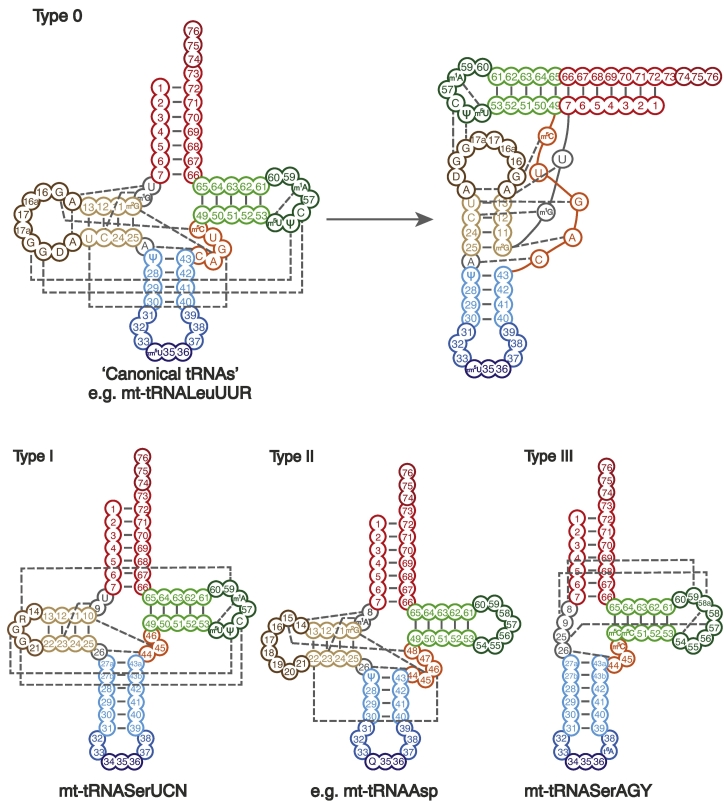
Tertiary interactions involved in the folding of mt-tRNAs. Schematic secondary structures and their tertiary interactions involved in tRNA folding are shown for canonical (type 0) along with three non-canonical folding patterns observed in mammalian mt-tRNAs (types I, II, and III). Example human mt-tRNAs written below their respective structures. A two-dimensional representation of the tertiary structure is presented for the canonical tRNA. Residues and modifications that are involved in tertiary interactions and well conserved among mammals are annotated in place of usual tRNA numbering. Acceptor stem in red, D-arm in brown, T-arm in green, variable loop in orange, and anticodon arm in blue. Tertiary interactions represented as dashed grey lines.

Following transcription and endonucleolytic processing, the canonical tRNA of around 75–90 nucleotides folds into a cloverleaf secondary structure due to the annealing of four base paired stems: the acceptor stem, T-stem, anticodon stem, and the D-stem, interconnected by three corresponding non-base paired loops: the T-loop, anticodon loop, and the D-loop. Interactions between distal residues within this cloverleaf structure, particularly between the D- and T-loops, bring the tRNA towards its tertiary structure of a compact L-shape, with its acceptor stem and anticodon stem perpendicular to one another and an ‘elbow’ between them (Fig. 4, type 0). For many tRNAs, including a number of mt-tRNAs, unmodified transcripts will naturally adopt the cloverleaf structure and are even translationally active *in vitro*, albeit with lower accuracy [121,122], others however are entirely dependent on core modifications to assist cloverleaf formation [123]. While a small number of mt-tRNAs, such as mt-tRNA^Leu(UUR)^ and mt-tRNA^Gln^, have retained the features associated with canonical folding [124], the majority are non-canonical relative to their nuclear-encoded counterparts. For example, although mt-tRNA^Ser(UCN)^ appears to have conserved D-loop/T-loop interaction, it displays a number of distinct structural features including an extended anticodon stem, a shortened D-loop, and only a single nucleotide separating the acceptor and D-stems (Fig. 4, type I). Despite these alterations, mt-tRNA^Ser(UCN)^ is still proposed to form a very similar tertiary structure [125]. The other mammalian serine isoacceptor, mt-tRNA^Ser(AGY)^, is also atypical, due to the absence of the entire D-arm (Fig. 4, type III). As a consequence of the D-arm's absence, mt-tRNA^Ser(AGY)^ has been shown to have a more flexible core region compared to canonical tRNAs, which allows it to match the tertiary structure dimensions accurately [126]. The remaining tRNAs that make up the majority operating within mammalian mitochondria lack many residues that are found to be well conserved outside of mitochondria, particularly G18, G19, Ψ55, and C56, that are required for the stabilising D-loop/T-loop interaction (Fig. 4, type II). Enzymatic and chemical probing of bovine mt-tRNA^Phe^ has suggested that these tRNAs rely entirely on interactions between the D-stem and the variable loop (V-loop) in order to achieve the L-shaped tertiary structure required [127]. Although, with the aid of post-transcriptional modifications, all of these tRNAs form structures optimised for translational efficiency and fidelity, it is important to consider this process as a structural dynamic equilibrium, with a multitude of alternative structural states coexisting, and the modifications discussed here shifting a particular structure towards the predominant form. While there are examples of modifications that cause large shifts due to significant rearrangement of base-pairing [128], the majority of core modifications work within an intrinsic network, with any single modification contributing only a fine-tuning in flexibility or rigidity [129].

#### 3.1.1 Methylations in the tRNA core

Core modifications are typically simpler in their chemistry compared to functional modifications, consisting largely of methylations at various positions on the RNA base. Methylations on the Watson-Crick edge, and also those on the Hoogsteen edge and sugar edge, interfere with the formation of hydrogen bonds between nucleotides, and thereby block the formation of helices (*i.e.* stems within tRNAs) between certain RNA regions, and promote the formation of others. In this manner, large scale rearrangements of tRNAs by a core modification may be achieved. Unmodified human mt-tRNA^Lys^ exists in an equilibrium between a cloverleaf secondary structure and a non-functional hairpin structure, with the latter as the predominant form [130]. A single methylation of N1 in the adenosine residue at position 9 (m^1^A9), performed by a subcomplex of RNase P composed of MRPP1 (also known as TRMT10C) and MRPP2 [131], triggers the stabilisation of the cloverleaf fold by blocking the base pairing between A9 and U64 in the T-stem [128]. The m^1^A9 modification, also existing as m^1^G9 in some mt-tRNAs, is very rarely found in tRNAs operating outside of mitochondria, and yet highly abundant in mt-tRNAs (present in ~60% of bovine mt-tRNAs) where it may have arisen as an adaptation to a changing mitochondrial genome. However, the inverse is true for the same modification located at position 58 (m^1^A58), frequently absent in mitochondrial tRNAs (and curiously substoichiometric in the case of mt-tRNALys [132]) despite being a near ubiquitous modification in cytosolic tRNAs, where it is proposed to confer a positive charge to the tRNA elbow and thereby stabilise the tertiary structure [133]. TRMT61B is responsible for the formation of m^1^A58 in human mt-tRNAs [75], with the resulting modification proposed to be reversible through the demethylation activity of ALKBH1 [134], thereby allowing dynamic control over two tRNA fractions which may play a role in regulating the rate of translation. Although less is known about other methylations on the Watson-Crick edge in mt-tRNAs, both m^2^G10, predicted to be catalysed by the TRMT11/TRMT112 heterodimer, and m^2^G/m^2^_2_G26, shown to be catalysed by TRMT1 [135], have been identified in human mt-tRNAs [54]. The dimethylation to form m^2^_2_G26 was one of the first RNA modifications to be identified [136] and blocks base pairing with cytosine residues and so may function in a similar manner to m^1^A9 by interfering with unfavourable interactions [137]. A recent computational approach has demonstrated a decreased structural stability upon the loss of m^2^G or m^2^_2_G [138], moreover human cells deficient in TRMT1 activity display perturbed mitochondrial translation and hypersensitivity to oxidising agents [135]. Large scale structural rearrangements can also be induced through modifications that do not alter base pairing. Modifications made to tRNAs do not exert their effects in isolation, and are instead optimised to the interacting partners and ionic environment around them. The latter is demonstrated by the observation that the absence of certain modifications can be compensated for by an increase in the concentration of magnesium ions, indicating that tRNA modifications are modulated by magnesium ions in physiological conditions to produce optimal tRNA folding [139]. The presence of magnesium ions shields adjacent phosphates in the RNA backbone, allowing for a greater degree of compaction required for tertiary interactions [133]. Core modifications, such as m^5^C, have been shown to indirectly influence tRNA structure by improving the binding of magnesium ions [[140], [141], [142]].

#### 3.1.2 Other modifications in the tRNA core

As mentioned, the majority of the core modifications do not influence tRNA structure through the interference with Watson-Crick base pairing. Instead, the remainder exert their subtle influence within local RNA structure through either restricting or increasing conformational freedom, inducing structural rigidity or flexibility, respectively. These conformational shifts often present themselves as differences in the ‘sugar pucker’ of the ribose. Within unstructured RNA, the sugar conformation exists as an equilibrium between two major forms, the C2′-endo and the C3′-endo, with the distances between adjacent phosphorus atoms, and their orientation relative to the base, being dramatically different. The hybridisation of RNA into helices however, results in a shift to the 3′-endo conformation as the predominant form, and as a consequence, any nucleotide modifications that favour the 3′-endo form will act to stabilise the helix [143]. Furthermore, the relative geometry between the 2′-hydroxyl and the phosphate granted by the 3′-endo sugar pucker is found to protect the structure from spontaneous hydrolysis [144]. Such an effect is also reproduced through the isomerisation of uridine, forming pseudouridine (Ψ), the single most abundant modification found in tRNAs. While uracil is linked to the ribose at the N-1 position, isomerisation results in a rotated base linked to the ribose at the C-5 position, in a process that grants Ψ an additional hydrogen bond donor on its Hoogsteen edge in the form of the now available N1—H. The decrease in flexibility of local RNA structure is proposed to occur through the coordination of a water molecule between the N1—H of Ψ and the two phosphates to the 3′ and 5′, with the resulting water bridge restricting both base conformation and RNA backbone flexibility [145]. Members of the pseudouridine synthase family which carry out the isomerisation of uridine, exhibit a high degree of specificity for their target residue. As a consequence, many members of this family are typically expressed in any given organism in order to produce the high number of Ψ residues found within RNAs. PUS1 is by far the most well characterised pseudouridine synthase operating in human mitochondria, responsible for Ψ27 and Ψ28 in mt-tRNAs [146]. Although many further pseudouridine sites exist within human mt-tRNAs, and candidate enzymes for their formation have been identified [76], demonstration of their role in human mt-tRNAs is still lacking. Conversely, uridine residues may also be modified in a manner that allows for greater conformational flexibility through the saturation of the pyrimidine ring, producing dihydrouridine (D). The absence of the C5=C6 double bond results in a non-planar base that resists stacking and promotes the 2′-endo sugar conformation [147]. Dihydrouridine is found in position 20 in the D-arm of mt-tRNAs, with the reduction of uracil predicted to be performed by DUS2 in humans. The impact of the above modifications is exemplified in the case of extremophiles, where the functional optimum flexibility must be maintained in species whose environment may be in excess of 100 °C or lower than 0 °C. Thermophiles employ a wide range of RNA modifications to increase tRNA stability such as ac^4^Cm (N4-acetyl-2′-*O*-methylcytidine), s^2^T (5-methyl-2-thiouridine), and m^1^A, which can be induced by high temperatures [148,149]. On the other end of the scale, in psychrophilic bacteria with an optimal growth temperature of below 15 °C, tRNAs are found to have a significantly higher presence of dihydrouridines in order to increase their flexibility [150]. All of these modifications are clustered within the nucleotide regions governing D- and T-loop interactions, underpinning the importance of these two domains in the stability of tertiary structure.

### 3.2 Functional modifications

The L-shaped tertiary folding of a tRNA brings the two ‘functional sites’ - the anticodon loop and the acceptor stem - into correct orientation. In effect, these two sites represent the points of translation between the mRNA triplet code and protein sequence, and modifications at both are crucial for the accurate flow of genetic information between the two.

The anticodon loop, particularly positions 34 and 37, is a hotspot of post-transcriptional modifications that play a key role in maintaining translation fidelity and accuracy by modulating the interaction between the tRNA and mRNA. One of both of these modifications is found to be modified in almost every tRNA studied, with the modifications present representing the greatest degree of chemical diversity found within tRNAs.

#### 3.2.1 Wobble base modifications

The translation of an mRNA into its corresponding polypeptide chain is dependent on the precise interactions between the three bases (referred to as 1, 2 and 3, numbered 5′ to 3′) of the mRNA's triplet codon and the triplet anticodon of the cognate tRNA (at positions 36, 35 and 34). However, owing to degeneracy of the genetic code, multiple codons must be recognized by a single tRNA, a requirement that is particularly evident in mitochondria as a product of the highly reduced mtDNA, resulting in single tRNAs responsible for the decoding on an entire codon box. Degenerate codons contain identical residues in positions 1 and 2 and are expanded through variability in position 3. To accomplish this, the interactions between residues 3 and 34 are non-standard, allowing for a much greater range of possible base pairs, a characteristic referred to as ‘wobble’. Position 34, or the wobble base, is often occupied by a uridine, capable of base pairing with any of the four bases due to enhanced conformational flexibility within the anticodon loop [151]. This scenario is sufficient for the majority of codons in which the residue in position 3 is entirely degenerate. However, in a number of cases, the presence of a purine or a pyrimidine in position 3 produces codons for different amino acids. The increase in discrimination by the wobble base required for accurate decoding is achieved through its post-transcriptional modification [152,153]. For example, the formation of the τm^5^U34 modification by the concerted action of GTPBP3 (GTP binding protein 3) [154], MTO1 (mitochondrial tRNA translation optimisation 1) [51,155], and the further thiolation of a subset of substrates by MTU1 (mitochondrial tRNA-specific 2-thiouridylase, TRMU) to form τm^5^(s^2^)U34 [156], greatly favours base pairing with purines and prevents codon misreading [157] as has been demonstrated for human mt-tRNA^Leu(UUR)^ [158]. The recognition of purines at position 3 is also exemplified by the formylation in mt-tRNA^Met^ which assists mt-tRNAs in the translation of the slightly modified genetic code compared to the cytosol. In the cytosol, a single codon, AUG, encodes for methionine and is recognized by two different tRNAs, one for initiation and one for elongation [159]. In mitochondria, however, methionine encoding is expanded to AUA, as well as AUG, with both codons being recognized by a single tRNA bearing a CAU anticodon [160]. The modification of C34 to f^5^C34 is believed to expand the codon recognition capabilities of mt-tRNA^Met^ through enhanced binding to AUA [161,162]. The biogenesis of f^5^C34 is initiated by a methylation forming m^5^C34 by NSUN3 [48,52], which is subsequently oxidised to f^5^C34 by ABH1 [134,163]. The status of m^5^C34 modified mt-tRNA^Met^ *in vivo*, either simply as a reaction intermediate or as a functionally separate population of tRNAs, remains uncertain [164]. The final modified base thus far found at position 34 of human mitochondrial tRNAs, queuosine (Q), represents an interesting case in which rather than an encoded base being modified *in situ*, the entire base (in this case a guanine) is excised and replaced through a breakage of the glycosidic bond. The substitution of guanine for Q is performed by tRNA-guanine transglycosylases (TGTases), with QTRTD1, one of two mammalian TGTases, believed to perform the reaction in human mitochondria [165,166]. As with previous wobble base modifications, Q34 has been implicated in modifying the decoding capabilities of a tRNA [167].

#### 3.2.2 Position 37

While stringent selection of the correct cognate tRNA is key to ensuring accuracy, a stable codon-anticodon interaction is critical for translation efficiency. For this reason, tRNAs with anticodons bearing U and A in position 36 often require modification at the adjacent position 37 [152,168]. For example, human mt-tRNAs contain a highly evolutionarily conserved *N*^6^-isopentenyladenosine (i^6^A37) catalysed by TRIT1 (tRNA isopentenyltransferase 1) [169] which in some cases is further modified by methylthiolation to ms^2^i^6^A37 by CDK5RAP1 (Cyclin-dependent kinase 5 regulatory subunit associated protein 1) [170] which acts to stabilise the intrinsically weak A–U pairing between A36 in the anticodon and position 1 of UNN codons. Work in yeast has demonstrated that i^6^A37 promotes translational efficiency and fidelity by granting a roughly four-fold increase in the specific activity of a tRNA for its codon [171]. In human mt-tRNAs, A37 is also modified to *N*^6^-threonylcarbamoyl adenosine (t^6^A), which has also been demonstrated to play a critical role in maintaining decoding accuracy [172]. Its biosynthesis in humans depends on the activities of YRDC and OSGEPL1, acting on their substrates L-threonine, ATP, and CO_2_/bicarbonate [173]. The availability of CO_2_ has been suggested to be the rate limiting factor is t^6^A37 biosynthesis, leading to a proposal of codon specific translational regulation in response to intracellular CO_2_ concentrations. Although adenosine is its most common identity, a guanosine residue may also be present at position 37 in mammalian mt-tRNAs, which is often methylated to form *N*^1^-methylguanosine (m^1^G37). This modification is found to be critical for tRNAs reading CNN codons, where the methylation at N1 blocks Watson-Crick base pairing between G37 and C1 within the codon. The absence of m^1^G37 allows base pairing to occur with C1 of the codon, resulting in a four nucleotide codon-anticodon interaction and a shift in the reading frame of the mRNA. This +1 frameshifting is highly deleterious to the cell, often causing a premature stop codon and the release of a potentially toxic truncated polypeptide [174,175]. In addition to the prevention of frameshifting, the loss of m^1^G at position 37 has also been linked to a reduced stringency on aminoacyl-tRNA selection at the ribosome [176], a reduced rate of polypeptide elongation [177], and an increase in the misacylation of the tRNA [178,179]. The methylation is performed in humans by TRMT5 [50]. As in the core of the tRNA, the anticodon loops from many species, including humans, have been found to contain modifications known to modulate local RNA flexibility, thus assisting the loop conformation required for interacting with the mRNA. For example, pseudouridine has been identified in human mt-tRNAs at positions 31, 32, and 39, with the latter demonstrated to be introduced by the pseudouridine synthetase RPUSD4 [99].

#### 3.2.3 Modifications at the termini of mitochondrial tRNAs

The acceptor stem acts as the amino acid attachment site, through its esterification onto either the 2′- or 3′-hydroxyl of the 3′-terminal residue, however this cannot occur on the 3′-terminus generated through RNase Z activity, and is instead dependent on its post-transcriptional modification. A universally conserved CCA sequence is added to all tRNAs in a non-templated post-transcriptional polymerisation event, performed in human mitochondria by the essential enzyme TRNT1 (tRNA-nucleotidyltransferase 1) [20], with the final A acting as the attachment site. Additionally, studies on *in vitro* transcribed tRNAs have reported that the CCA sequence acts as a tRNase Z anti-determinant, ensuring that a futile cycle between polymerase and ribonuclease activities does not occur [180]. In cytosolic tRNAs, the CCA sequence existing in the form of a tandem CCACCA on the 3′ end has been identified as a quality control signal, targeting misfolded or hypomodified tRNAs for degradation [181], however no such pathway has been demonstrated in mitochondria. The 3′ ends of several mt-tRNAs are spuriously polyadenylated precluding correct aminoacylation at the 3′ end (see below). A 3′–5′ exoribonuclease, PDE12, is required for the removal of these aberrant poly(A) tails [22,182]. In almost all species, a single tRNA, tRNA^His^, is also modified at the 5′ end with an additional guanosine, termed G_−1_ [183], which acts as a recognition determinant for its cognate histidyl-tRNA synthetase [184]. Although the presence of G_−1_ has been shown to result from an altered RNase P cleavage of an encoded guanosine in bacteria [185] and yeast mitochondria [186], G_−1_ is not encoded in human mtDNA and its post-transcriptional addition on human mt-tRNAHis by THG1L has been demonstrated *in vitro* [187].

The esterification of the 3′-CCA end is performed by a family of aminoacyl-tRNA synthetases (aaRSs), with each member highly specific to both a particular amino acid and its recognition of the cognate tRNA or family of isoacceptors. The recognition of a particular tRNA by its cognate aaRS is governed by identity elements present within the tRNA, with the majority located in either anticodon stem (especially the discriminator base, N73) or the anticodon loop, although identity elements may also be found in the tRNA core [188]. Particular sets of identity elements are found to be conserved for a given amino acid even across the different kingdoms of life, however in many cases this consistency is found to not extend to mammalian mitochondria. As a case in point: G73 in the acceptor stem, G10 in the D-stem, and the anticodon triplet G34-U35-C36, together represent an identification pattern that is highly conserved throughout evolution in the aspartylation of tRNAs, with the transposition of these residues into another tRNA sufficient to convert it into an aspartic acid acceptor [189]. However, through the mutagenic analysis of human mt-tRNA^Asp^, only U35 and C36 were found to be critical for the recognition and aspartylation by its corresponding synthetase (DARS2), while changes of the other elements were found to have no effect on aminoacylation [190]. This striking deviation from recognition elements previously held to be universal emphasises both the significant divergence of mt-tRNAs from their canonical counterparts, and the evolutionary adaptation of the nuclear encoded synthetase. For 17 of the 19 aaRS operating within mitochondria, they are encoded on separate genes distinguishing them from the cytosolic synthetases, with only the synthetases for lysine and glycine encoded on the same gene, with differing protein products as a result of alternative splicing and alternative translation initiation, respectively [191,192]. The remaining exception concerns mt-tRNA^Gln^, for which there is no specific synthetase in mitochondria. Instead, mt-tRNA^Gln^ is correctly esterfied through the initial misaminoacylation with glutamate by EARS2 followed by the transamidation of esterfied glutamate to form glutamine by the GatCAB complex [193,194]. Finally, in the case of mt-tRNAMet, following the aminoacylation with methionine, the amino acid itself is further modified through a formylation performed by MTFMT, which increases its affinity towards the mitochondrial initiation factor (IF2_mt_) [195].

## 4 Mitochondrial mRNA modifications

Historically, the post-transcriptional maturation of mammalian mitochondrial mRNAs has been limited to endonucleolytic excision from the precursor polycistronic transcript and polyadenylation [71,196]. However, reports are emerging describing the presence of modified nucleotides in mt-mRNA and suggesting that epitranscriptomic regulation may play a role in fine-tuning mitochondrial gene expression and translation.

Transcriptome-wide pseudouridine profiling suggested that, in addition to the known pseudouridine sites in 16S mt-rRNA [97] and mt-tRNA [54], specific mt-mRNAs are pseudouridylated in human cells [[197], [198], [199], [200]]. However, confirmation by a more targeted approaches or characterisation of the enzymatic machinery has been missing. Recent identification of several mitochondrial putative pseudouridine synthases (PUS) (PUS1L, TRUB2, RPUSD3 and RPUSD4), allowed for more detailed studies of the role of pseudouridylation in mammalian mitochondria by using reverse transcription primer extension or next generation sequencing-based pseudouridine analysis (ψ-Seq) in cells depleted in the mitochondrial PUS enzymes [[98], [99], [100]]. As described in the previous sections, these studies reported RPUSD4 as a modifier of 16S mt-rRNA and mt-tRNAs (2.1.5, 3.2.2). In contrast, depletion of TRUB2 and RPUSD3 has been associated with decreased pseudouridylation in two mitochondrial mRNAs: MT-CO1 and MT-CO3. Notably, the presence of the previously suggested ψ in MT-COII and MT-ND4 mt-mRNAs [197] has not been confirmed in the later study by Antonicka et al. [100]. Therefore, a more targeted validation of the previous finding is required, ideally in the context of PUS gene knock-outs, to examine the role of mt-mRNA pseudouridylation in the regulation of mtDNA-encoded genes.

The presence of m^1^A in mt-mRNAs has been recently shown by two independent studies that mapped this modification at single resolution [201,202]. These two studies used next generation sequencing approach and analysed misincorporation patterns introduced by reverse transcription of m^1^A modified RNA [203]. The specificity and stringency of the approaches were additionally verified by comparing the misincorporations patters upon enrichment of modified RNA with anti-m^1^A antibodies and in chemically or enzymatically demethylated samples. In addition, Safra et al. reported an approach based upon a reverse transcriptase that stalls on m^1^A, leading to premature truncation of DNA products [202]. Both studies detected the previously reported m^1^A in mt-tRNA and mt-rRNA [72,75], confirming the value of these novel methods in detecting m^1^A. Importantly, previously unreported m^1^A sites have been detected in several mt-mRNAs, however, the two studies did not agree in respect of their number, location and stoichiometry; about 50 sites were detected in several sense and antisense mt-mRNAs by [201], whereas only 5 sites in 5 different messengers were reported by [202] (the reasons for this discrepancy is discussed in detail in [204]). Nonetheless, in both cases the MT-ND5 mRNA was reported as frequently modified, with most transcripts containing internal m^1^A (MT-ND5: A1374, mtDNA: m.13710A), concluding that this internal modification position in mRNA represses translation. In addition, Safra et al. studied the frequency of m^1^A in MT-ND5 in developing human embryos, observing that in oocytes and early embryos (up to the four-cell stage), nearly all MT-ND5 mRNAs are modified, whereas later developmental stages showed substantially reduced levels of this modification. The m^1^A in MT-ND5:1374 was also suggested to depend on TRMT10C, the enzyme previously shown to be a component of mitochondrial RNase P that introduces m^1^A (as well as m^1^G) at position 9 of mt-tRNA [131] (see previous sections). Moreover, they also found that if MT-ND5 contains a single nucleotide polymorphism (m.13708G > A), which is characteristic for mitochondrial haplogroup J and is linked to a synergistic and deleterious interaction with a mtDNA disease known as Leber's hereditary optic neuropathy (LHON), the formation of m^1^A in MT-ND5:1374 is reduced. Defects in m^1^A formation might therefore be linked to mitochondrial disease. On the other hand, Li et al. [201] reported that m^1^A sites located within the coding region of some, but not all m^1^A-harbouring mt-mRNAs (MT-CO1, MT-CO2, MT-CO3, MT-CYB, and MT-ND4L) depend on TRMT61B, which is responsible for m^1^A methylation at position 58 of mt-tRNAs and position 947 of 12S mt-rRNA [72,75]. While, these single-nucleotide resolution maps of m^1^A in mt-mRNA are expected to facilitate mechanistic studies of the function of m^1^A in mitochondrial RNA metabolism, several questions remain open. For example, how does m^1^A mediate mitochondrial translation inhibition? How are *bone fide* house-keeping enzymes indispensable for mt-tRNA folding and endonucleolytic processing of mitochondrial precursors (TRMT10C, [16]) or well conserved structural modifications of mt-tRNA and mt-rRNA (TRMT61B) regulated for modifying only a subset of m^1^A in mt-mRNA? Further research will be required to understand this novel layer of complexity of mitochondrial gene expression provided by the epitranscriptomic modifications.

## 5 Concluding remarks and future directions

Post-transcriptional RNA modifications have been emerging as one of the key regulators of mitochondrial gene expression, with numerous studies on their role in human health and disease reported recently. However, many fundamental problems, often related to the molecular mechanisms of mitochondrial disease, remain unsolved and new issues have emerged.

One of the next, more immediate goals for the mammalian mitochondrial epitranscriptomic field would be to continue the identification of post-transcriptional mtRNA modifications and enzymes responsible for their introduction. Surely, the recently developed high-throughput, next-generation sequencing-based, single nucleotide resolution methods and ever more sensitive mass spectrometry approaches for RNA will be of great help in this endeavour. This could be followed by reverse genetics approaches to understand which enzymes introduce particular modifications. Armed with this, functional investigations of these modifications will be possible to assess their effect on mtRNA stability, mitoribosome biogenesis, translation, and processing.

Regarding the role of RNA modifications in the mitoribosome, in addition to a more complete characterisation of structure-function relationship of the mt-rRNA modifications, it would be useful to assess possible roles of the enzymes involved in the deposition of such modifications in the biogenesis process of these macromolecular complexes. This hypothesis has been proposed before on several occasions [45,80,102], and takes special relevance due to the functional and overall structural conservation of mitoribosomes *versus* ribosomes of other compartments and species, especially because the mitochondrial proteome is more restricted in number and diversity of its components. Furthermore, with the advent and optimisation of high throughput, transcriptome-wide techniques for the targeted detection of RNA modifications, as well as cryoEM [205], it will be now possible to dive to greater depths of biological detail and identify novel modified residues in mt-rRNA. For instance, in addition to the well-established aforementioned residues (Section 2), several novel m^1^A sites were recently reported for mt-rRNAs, which need to be given attention, not only in terms of verification, but also regarding the enzymes involved in their deposition and their functional relevance [201].

Owing to the dual genetic origin of OxPhos complexes, the translation of nucleus-encoded and mtDNA-derived components needs to be coordinated to prevent deficiency or excess of subunits during the assembly of OxPhos complexes in the mitochondrial inner membrane. Recent studies in yeast indicated that such a coordination occurs at translation, rather than, transcription level. Several enzymes responsible for epitranscriptomic modifications of mtRNA have been also shown to localise outside of mitochondria and modify cytoplasmic RNA targets. These include the factors that modify both cytoplasmic and mitochondrial tRNAs at position 37 such like TRIT1 (i^6^A37), CDK5RAP1 (ms^2^i^6^A37) and YRDC (t^6^A37). Is it possible, therefore, that regulatory mechanisms exist to coordinate mitochondrial and cytosolic translation which operate at the level of the epitranscriptome?

In addition to their role in generating ATP through OxPhos, mitochondria are also metabolic hubs of the cell, hosting numerous biosynthetic pathways. The functional groups used in many post-transcriptional RNA modifications in mitochondria are derived from these pathways, while others are sourced from outside of mitochondria. For example, wobble 5-taurinomethyluridine (τm^5^U34) found in several mt-tRNAs, depends on 5,10-methylene-tetrahydrofolate (THF), the product of one‑carbon (1C) metabolism partially hosted by mitochondria, and on taurine sourced outside of mitochondria. Depletion of mitochondrial 5,10-methylene-THF by inactivation of serine hydroxymethyltransferase 2 (SHMT2) or taurine starvation decreases the levels of τm^5^U34 [51,206]. However, in the latter condition, cmnm^5^U34, in which the taurine moiety of τm5U is replaced with glycine, can be observed in mt-tRNAs [51]. This indicates that mt-tRNA modifications can be dynamically regulated in response to metabolic status impacting on mitochondrial translation in a codon-specific manner. It would be fascinating to explore if other mtRNA modifications are wired with cellular metabolism and the regulatory role of such interdependency.

Finally, functional studies of the mitochondrial transcriptome (and on mechanisms regulating mtDNA gene expression in general) are hindered by our inability to edit the mitochondrial genome, with site-directed mutagenesis of sequences coding for modified nucleotides in mtRNA being currently impossible [207,208]. Also, the development of an *in vitro* system for mitochondrial translation would be useful to directly establish the mechanistic details of the role of modified ribonucleotides in mitochondrial translation. Resolving these issues would enable a more comprehensive approach for functional investigations on the mammalian mitochondrial epitranscriptome.

## Transparency document

Transparency document Image 1

## References

[1] Gray M.W., Burger G., Lang B.F. (1999). Mitochondrial evolution. Science.

[2] Anderson S., Bankier A.T., Barrell B.G., de Bruijn M.H., Coulson A.R., Drouin J., Eperon I.C., Nierlich D.P., Roe B.A., Sanger F., Schreier P.H., Smith A.J., Staden R., Young I.G. (1981). Sequence and organization of the human mitochondrial genome. Nature.

[3] Fearnley I.M., Walker J.E. (1986). Two overlapping genes in bovine mitochondrial DNA encode membrane components of ATP synthase. EMBO J..

[4] Tiranti V., Savoia A., Forti F., D'Apolito M.F., Centra M., Rocchi M. Zeviani (1997). Identification of the gene encoding the human mitochondrial RNA polymerase (h-mtRPOL) by cyberscreening of the expressed sequence tags database. Hum. Mol. Genet..

[5] Kukat C., Davies K.M., Wurm C.A., Spahr H., Bonekamp N.A., Kuhl I., Joos F., Polosa P.L., Park C.B., Posse V., Falkenberg M., Jakobs S., Kuhlbrandt W., Larsson N.G. (2015). Cross-strand binding of TFAM to a single mtDNA molecule forms the mitochondrial nucleoid. Proc. Natl. Acad. Sci. U. S. A..

[6] Falkenberg M., Gaspari M., Rantanen A., Trifunovic A., Larsson N.G., Gustafsson C.M. (2002). Mitochondrial transcription factors B1 and B2 activate transcription of human mtDNA. Nat. Genet..

[7] McCulloch V., Seidel-Rogol B.L., Shadel G.S. (2002). A human mitochondrial transcription factor is related to RNA adenine methyltransferases and binds S-adenosylmethionine. Mol. Cell. Biol..

[8] Posse V., Gustafsson C.M. (2017). Human mitochondrial transcription factor B2 is required for promoter melting during initiation of transcription. J. Biol. Chem..

[9] Seidel-Rogol B.L., McCulloch V., Shadel G.S. (2003). Human mitochondrial transcription factor B1 methylates ribosomal RNA at a conserved stem-loop. Nat. Genet..

[10] Sologub M., Litonin D., Anikin M., Mustaev A., Temiakov D. (2009). TFB2 is a transient component of the catalytic site of the human mitochondrial RNA polymerase. Cell.

[11] Hillen H.S., Morozov Y.I., Sarfallah A., Temiakov D., Cramer P. (2017). Structural basis of mitochondrial transcription initiation. Cell.

[12] Minczuk M., He J., Duch A.M., Ettema T.J., Chlebowski A., Dzionek K., Nijtmans L.G., Huynen M.A., Holt I.J. (2011). TEFM (c17orf42) is necessary for transcription of human mtDNA. Nucleic Acids Res..

[13] Agaronyan K., Morozov Y.I., Anikin M., Temiakov D. (2015). Mitochondrial biology. Replication-transcription switch in human mitochondria. Science.

[14] Posse V., Shahzad S., Falkenberg M., Hallberg B.M., Gustafsson C.M. (2015). TEFM is a potent stimulator of mitochondrial transcription elongation in vitro. Nucleic Acids Res..

[15] Ojala D., Montoya J., Attardi G. (1981). tRNA punctuation model of RNA processing in human mitochondria. Nature.

[16] Holzmann J., Frank P., Loffler E., Bennett K.L., Gerner C., Rossmanith W. (2008). RNase P without RNA: identification and functional reconstitution of the human mitochondrial tRNA processing enzyme. Cell.

[17] Brzezniak L.K., Bijata M., Szczesny R.J., Stepien P.P. (2011). Involvement of human ELAC2 gene product in 3′ end processing of mitochondrial tRNAs. RNA Biol..

[18] Rossmanith W. (2011). Localization of human RNase Z isoforms: dual nuclear/mitochondrial targeting of the ELAC2 gene product by alternative translation initiation. PLoS One.

[19] Sanchez M.I., Mercer T.R., Davies S.M., Shearwood A.M., Nygard K.K., Richman T.R., Mattick J.S., Rackham O., Filipovska A. (2011). RNA processing in human mitochondria. Cell Cycle.

[20] Nagaike T., Suzuki T., Tomari Y., Takemoto-Hori C., Negayama F., Watanabe K., Ueda T. (2001). Identification and characterization of mammalian mitochondrial tRNA nucleotidyltransferases. J. Biol. Chem..

[21] Wedatilake Y., Niazi R., Fassone E., Powell C.A., Pearce S., Plagnol V., Saldanha J.W., Kleta R., Chong W.K., Footitt E., Mills P.B., Taanman J.W., Minczuk M., Clayton P.T., Rahman S. (2016). TRNT1 deficiency: clinical, biochemical and molecular genetic features. Orphanet J. Rare Dis..

[22] Pearce S.F., Rorbach J., Van Haute L., D'Souza A.R., Rebelo-Guiomar P., Powell C.A., Brierley I., Firth A.E., Minczuk M. (2017). Maturation of selected human mitochondrial tRNAs requires deadenylation. elife.

[23] Antonicka H., Sasarman F., Nishimura T., Paupe V., Shoubridge E.A. (2013). The mitochondrial RNA-binding protein GRSF1 localizes to RNA granules and is required for posttranscriptional mitochondrial gene expression. Cell Metab..

[24] Antonicka H., Shoubridge E.A. (2015). Mitochondrial RNA granules are centers for posttranscriptional RNA processing and ribosome biogenesis. Cell Rep..

[25] Jourdain A.A., Koppen M., Rodley C.D., Maundrell K., Gueguen N., Reynier P., Guaras A.M., Enriquez J.A., Anderson P., Simarro M., Martinou J.-C. (2015). A mitochondria-specific isoform of FASTK is present in mitochondrial RNA granules and regulates gene expression and function. Cell Rep..

[26] Jourdain A.A., Koppen M., Wydro M., Rodley C.D., Lightowlers R.N., Chrzanowska-Lightowlers Z.M., Martinou J.C. (2013). GRSF1 regulates RNA processing in mitochondrial RNA granules. Cell Metab..

[27] Tu Y.T., Barrientos A. (2015). The human mitochondrial DEAD-box protein DDX28 resides in RNA granules and functions in mitoribosome assembly. Cell Rep..

[28] Jourdain A.A., Boehm E., Maundrell K., Martinou J.C. (2016). Mitochondrial RNA granules: compartmentalizing mitochondrial gene expression. J. Cell Biol..

[29] Ott M., Amunts A., Brown A. (2016). Organization and regulation of mitochondrial protein synthesis. Annu. Rev. Biochem..

[30] D'Souza A.R., Minczuk M. (2018). Mitochondrial transcription and translation: overview. Essays Biochem..

[31] Mai N., Chrzanowska-Lightowlers Z.M., Lightowlers R.N. (2017). The process of mammalian mitochondrial protein synthesis. Cell Tissue Res..

[32] Boccaletto P., Machnicka M.A., Purta E., Piatkowski P., Baginski B., Wirecki T.K., de Crecy-Lagard V., Ross R., Limbach P.A., Kotter A., Helm M., Bujnicki J.M. (2018). MODOMICS: a database of RNA modification pathways. 2017 update. Nucleic Acids Res..

[33] Jackman J.E., Alfonzo J.D. (2013). Transfer RNA modifications: nature's combinatorial chemistry playground. Wiley Interdiscip. Rev. RNA.

[34] Gilbert W.V., Bell T.A., Schaening C. (2016). Messenger RNA modifications: form, distribution, and function. Science.

[35] Chawla M., Oliva R., Bujnicki J.M., Cavallo L. (2015). An atlas of RNA base pairs involving modified nucleobases with optimal geometries and accurate energies. Nucleic Acids Res..

[36] Motorin Y., Helm M. (2011). RNA nucleotide methylation. Wiley Interdiscip. Rev. RNA.

[37] Kawai G., Yamamoto Y., Kamimura T., Masegi T., Sekine M., Hata T., Iimori T., Watanabe T., Miyazawa T., Yokoyama S. (1992). Conformational rigidity of specific pyrimidine residues in tRNA arises from posttranscriptional modifications that enhance steric interaction between the base and the 2′-hydroxyl group. Biochemistry.

[38] Lustig F., Boren T., Claesson C., Simonsson C., Barciszewska M., Lagerkvist U. (1993). The nucleotide in position 32 of the tRNA anticodon loop determines ability of anticodon UCC to discriminate among glycine codons. Proc. Natl. Acad. Sci. U. S. A..

[39] Davis F.F., Allen F.W. (1957). Ribonucleic acids from yeast which contain a fifth nucleotide. J. Biol. Chem..

[40] Cohn W.E., Volkin E. (1951). Nucleoside-5′-phosphates from ribonucleic acid. Nature.

[41] Cohn W.E. (1960). Pseudouridine, a carbon-carbon linked ribonucleoside in ribonucleic acids: isolation, structure, and chemical characteristics. J. Biol. Chem..

[42] Karijolich J., Yi C., Yu Y.T. (2015). Transcriptome-wide dynamics of RNA pseudouridylation. Nat. Rev. Mol. Cell Biol..

[43] Charette M., Gray M.W. (2000). Pseudouridine in RNA: what, where, how, and why. IUBMB Life.

[44] Pearce S.F., Rebelo-Guiomar P., D'Souza A.R., Powell C.A., Van Haute L., Minczuk M. (2017). Regulation of mammalian mitochondrial gene expression: recent advances. Trends Biochem. Sci..

[45] Sirum-Connolly K., Peltier J.M., Crain P.F., McCloskey J.A., Mason T.L. (1995). Implications of a functional large ribosomal RNA with only three modified nucleotides. Biochimie.

[46] Decatur W.A., Fournier M.J. (2002). rRNA modifications and ribosome function. Trends Biochem. Sci..

[47] Torres A.G., Batlle E., Ribas De Pouplana L. (2014). Role of tRNA modifications in human diseases. Trends Mol. Med..

[48] Van Haute L., Dietmann S., Kremer L., Hussain S., Pearce S.F., Powell C.A., Rorbach J., Lantaff R., Blanco S., Sauer S., Kotzaeridou U., Hoffmann G.F., Memari Y., Kolb-Kokocinski A., Durbin R., Mayr J.A., Frye M., Prokisch H., Minczuk M. (2016). Deficient methylation and formylation of mt-tRNA(met) wobble cytosine in a patient carrying mutations in NSUN3. Nat. Commun..

[49] Garone C., D'Souza A.R., Dallabona C., Lodi T., Rebelo-Guiomar P., Rorbach J., Donati M.A., Procopio E., Montomoli M., Guerrini R., Zeviani M., Calvo S.E., Mootha V.K., DiMauro S., Ferrero I., Minczuk M. (2017). Defective mitochondrial rRNA methyltransferase MRM2 causes MELAS-like clinical syndrome. Hum. Mol. Genet..

[50] Powell C.A., Kopajtich R., D'Souza A.R., Rorbach J., Kremer L.S., Husain R.A., Dallabona C., Donnini C., Alston C.L., Griffin H., Pyle A., Chinnery P.F., Strom T.M., Meitinger T., Rodenburg R.J., Schottmann G., Schuelke M., Romain N., Haller R.G., Ferrero I., Haack T.B., Taylor R.W., Prokisch H., Minczuk M. (2015). TRMT5 mutations cause a defect in post-transcriptional modification of mitochondrial tRNA associated with multiple respiratory-chain deficiencies. Am. J. Hum. Genet..

[51] Asano K., Suzuki T., Saito A., Wei F.Y., Ikeuchi Y., Numata T., Tanaka R., Yamane Y., Yamamoto T., Goto T., Kishita Y., Murayama K., Ohtake A., Okazaki Y., Tomizawa K., Sakaguchi Y., Suzuki T. (2018). Metabolic and chemical regulation of tRNA modification associated with taurine deficiency and human disease. Nucleic Acids Res..

[52] Nakano S., Suzuki T., Kawarada L., Iwata H., Asano K., Suzuki T. (2016). NSUN3 methylase initiates 5-formylcytidine biogenesis in human mitochondrial tRNA(met). Nat. Chem. Biol..

[53] Van Haute L., Pearce S.F., Powell C.A., D'Souza A.R., Nicholls T.J., Minczuk M. (2015). Mitochondrial transcript maturation and its disorders. J. Inherit. Metab. Dis..

[54] Suzuki T., Nagao A., Suzuki T. (2011). Human mitochondrial tRNAs: biogenesis, function, structural aspects, and diseases. Annu. Rev. Genet..

[55] Bohnsack M.T., Sloan K.E. (2018). The mitochondrial epitranscriptome: the roles of RNA modifications in mitochondrial translation and human disease. Cell. Mol. Life Sci..

[56] Boczonadi V., Ricci G., Horvath R. (2018). Mitochondrial DNA transcription and translation: clinical syndromes. Essays Biochem..

[57] Powell C.A., Nicholls T.J., Minczuk M. (2015). Nuclear-encoded factors involved in post-transcriptional processing and modification of mitochondrial tRNAs in human disease. Front. Genet..

[58] Rorbach J., Gao F., Powell C.A., D'Souza A., Lightowlers R.N., Minczuk M., Chrzanowska-Lightowlers Z.M. (2016). Human mitochondrial ribosomes can switch their structural RNA composition. Proc. Natl. Acad. Sci. U. S. A..

[59] Brown A., Amunts A., Bai X.C., Sugimoto Y., Edwards P.C., Murshudov G., Scheres S.H.W., Ramakrishnan V. (2014). Structure of the large ribosomal subunit from human mitochondria. Science.

[60] Greber B.J., Boehringer D., Leitner A., Bieri P., Voigts-Hoffmann F., Erzberger J.P., Leibundgut M., Aebersold R., Ban N. (2014). Architecture of the large subunit of the mammalian mitochondrial ribosome. Nature.

[61] Amunts A., Brown A., Toots J., Scheres S.H.W., Ramakrishnan V. (2015). Ribosome. The structure of the human mitochondrial ribosome. Science.

[62] Bieri P., Greber B.J., Ban N. (2018). High-resolution structures of mitochondrial ribosomes and their functional implications. Curr. Opin. Struct. Biol..

[63] Spenkuch F., Motorin Y., Helm M. (2014). Pseudouridine: still mysterious, but never a fake (uridine)!. RNA Biol..

[64] Sergiev P.V., Aleksashin N.A., Chugunova A.A., Polikanov Y.S., Dontsova O.A. (2018). Structural and evolutionary insights into ribosomal RNA methylation. Nat. Chem. Biol..

[65] Sirum-Connolly K., Mason T. (1993). Functional requirement of a site-specific ribose methylation in ribosomal RNA. Science.

[66] Dubin D.T. (1974). Methylated nucleotide content of mitochondrial ribosomal RNA from hamster cells. J. Mol. Biol..

[67] Dubin D.T., Taylor R.H. (1978). Modification of mitochondrial ribosomal RNA from hamster cells: the presence of GmG and late-methylated UmGmU in the large subunit (17S) RNA. J. Mol. Biol..

[68] Dubin D.T., Taylor R.H., Davenport L.W. (1978). Methylation status of 13S ribosomal RNA from hamster mitochondria: the presence of a novel riboside, N4-methylcytidine. Nucleic Acids Res..

[69] Baer R., Dubin D.T. (1980). The 3′-terminal sequence of the small subunit ribosomal RNA from hamster mitochondria. Nucleic Acids Res..

[70] Baer R.J., Dubin D.T. (1981). Methylated regions of hamster mitochondrial ribosomal RNA: structural and functional correlates. Nucleic Acids Res..

[71] Rorbach J., Minczuk M. (2012). The post-transcriptional life of mammalian mitochondrial RNA. Biochem. J..

[72] Bar-Yaacov D., Frumkin I., Yashiro Y., Chujo T., Ishigami Y., Chemla Y., Blumberg A., Schlesinger O., Bieri P., Greber B., Ban N., Zarivach R., Alfonta L., Pilpel Y., Suzuki T., Mishmar D. (2016). Mitochondrial 16S rRNA is methylated by tRNA methyltransferase TRMT61B in all vertebrates. PLoS Biol..

[73] Bar-Yaacov D., Avital G., Levin L., Richards A.L., Hachen N., Rebolledo Jaramillo B., Nekrutenko A., Zarivach R., Mishmar D. (2013). RNA-DNA differences in human mitochondria restore ancestral form of 16S ribosomal RNA. Genome Res..

[74] Hodgkinson A., Idaghdour Y., Gbeha E., Grenier J.C., Hip-Ki E., Bruat V., Goulet J.P., de Malliard T., Awadalla P. (2014). High-resolution genomic analysis of human mitochondrial RNA sequence variation. Science.

[75] Chujo T., Suzuki T. (2012). Trmt61B is a methyltransferase responsible for 1-methyladenosine at position 58 of human mitochondrial tRNAs. RNA.

[76] Suzuki T., Suzuki T. (2014). A complete landscape of post-transcriptional modifications in mammalian mitochondrial tRNAs. Nucleic Acids Res..

[77] Arai T., Ishiguro K., Kimura S., Sakaguchi Y., Suzuki T., Suzuki T. (2015). Single methylation of 23S rRNA triggers late steps of 50S ribosomal subunit assembly. Proc. Natl. Acad. Sci. U. S. A..

[78] Ozanick S., Krecic A., Andersland J., Anderson J.T. (2005). The bipartite structure of the tRNA m1A58 methyltransferase from *S. cerevisiae* is conserved in humans. RNA.

[79] Anderson J., Phan L., Cuesta R., Carlson B.A., Pak M., Asano K., Bjork G.R., Tamame M., Hinnebusch A.G. (1998). The essential Gcd10p-Gcd14p nuclear complex is required for 1-methyladenosine modification and maturation of initiator methionyl-tRNA. Genes Dev..

[80] Lovgren J.M., Wikstrom P.M. (2001). The rlmB gene is essential for formation of Gm2251 in 23S rRNA but not for ribosome maturation in *Escherichia coli*. J. Bacteriol..

[81] Michel G., Sauve V., Larocque R., Li Y., Matte A., Cygler M. (2002). The structure of the RlmB 23S rRNA methyltransferase reveals a new methyltransferase fold with a unique knot. Structure.

[82] Bernardi G. (1979). The petite mutation in yeast. Trends Biochem. Sci..

[83] Struhl K. (1985). Nucleotide sequence and transcriptional mapping of the yeast pet56-his3-ded1 gene region. Nucleic Acids Res..

[84] Lee K.W., Bogenhagen D.F. (2014). Assignment of 2′-O-methyltransferases to modification sites on the mammalian mitochondrial large subunit 16 S ribosomal RNA (rRNA). J. Biol. Chem..

[85] Moazed D., Noller H.F. (1989). Intermediate states in the movement of transfer RNA in the ribosome. Nature.

[86] Bugl H., Fauman E.B., Staker B.L., Zheng F., Kushner S.R., Saper M.A., Bardwell J.C., Jakob U. (2000). RNA methylation under heat shock control. Mol. Cell.

[87] Caldas T., Binet E., Bouloc P., Costa A., Desgres J., Richarme G. (2000). The FtsJ/RrmJ heat shock protein of Escherichia coli is a 23 S ribosomal RNA methyltransferase. J. Biol. Chem..

[88] Tan J., Jakob U., Bardwell J.C.A. (2002). Overexpression of two different GTPases rescues a null mutation in a heat-induced rRNA methyltransferase. J. Bacteriol..

[89] Widerak M., Kern R., Malki A., Richarme G. (2005). U2552 methylation at the ribosomal A-site is a negative modulator of translational accuracy. Gene.

[90] Lavdovskaia E., Kolander E., Steube E., Mai M.M., Urlaub H., Richter-Dennerlein R. (2018). The human Obg protein GTPBP10 is involved in mitoribosomal biogenesis. Nucleic Acids Res..

[91] Kim D.F., Green R. (1999). Base-pairing between 23S rRNA and tRNA in the ribosomal A site. Mol. Cell.

[92] Myers A.M., Pape L.K., Tzagoloff A. (1985). Mitochondrial protein synthesis is required for maintenance of intact mitochondrial genomes in *Saccharomyces cerevisiae*. EMBO J..

[93] Pintard L., Bujnicki J.M., Lapeyre B., Bonnerot C. (2002). MRM2 encodes a novel yeast mitochondrial 21S rRNA methyltransferase. EMBO J..

[94] Rorbach J., Boesch P., Gammage P.A., Nicholls T.J., Pearce S.F., Patel D., Hauser A., Perocchi F., Minczuk M. (2014). MRM2 and MRM3 are involved in biogenesis of the large subunit of the mitochondrial ribosome. Mol. Biol. Cell.

[95] Lee K.W., Okot-Kotber C., Lacomb J.F., Bogenhagen D.F. (2013). Mitochondrial ribosomal RNA (rRNA) methyltransferase family members are positioned to modify nascent rRNA in foci near the mitochondrial DNA nucleoid. J. Biol. Chem..

[96] Bogenhagen D.F., Rousseau D., Burke S. (2008). The layered structure of human mitochondrial DNA nucleoids. J. Biol. Chem..

[97] Ofengand J., Bakin A. (1997). Mapping to nucleotide resolution of pseudouridine residues in large subunit ribosomal RNAs from representative eukaryotes, prokaryotes, archaebacteria, mitochondria and chloroplasts. J. Mol. Biol..

[98] Arroyo J.D., Jourdain A.A., Calvo S.E., Ballarano C.A., Doench J.G., Root D.E., Mootha V.K. (2016). A genome-wide CRISPR death screen identifies genes essential for oxidative phosphorylation. Cell Metab..

[99] Zaganelli S., Rebelo-Guiomar P., Maundrell K., Rozanska A., Pierredon S., Powell C.A., Jourdain A.A., Hulo N., Lightowlers R.N., Chrzanowska-Lightowlers Z.M., Minczuk M., Martinou J.C. (2017). The pseudouridine synthase RPUSD4 is an essential component of mitochondrial RNA granules. J. Biol. Chem..

[100] Antonicka H., Choquet K., Lin Z.Y., Gingras A.C., Kleinman C.L., Shoubridge E.A. (2017). A pseudouridine synthase module is essential for mitochondrial protein synthesis and cell viability. EMBO Rep..

[101] Demirci H., Larsen L.H., Hansen T., Rasmussen A., Cadambi A., Gregory S.T., Kirpekar F., Jogl G. (2010). Multi-site-specific 16S rRNA methyltransferase RsmF from *Thermus thermophilus*. RNA.

[102] Metodiev M.D., Spahr H., Loguercio Polosa P., Meharg C., Becker C., Altmueller J., Habermann B., Larsson N.G., Ruzzenente B. (2014). NSUN4 is a dual function mitochondrial protein required for both methylation of 12S rRNA and coordination of mitoribosomal assembly. PLoS Genet..

[103] Camara Y., Asin-Cayuela J., Park C.B., Metodiev M.D., Shi Y., Ruzzenente B., Kukat C., Habermann B., Wibom R., Hultenby K., Franz T., Erdjument-Bromage H., Tempst P., Hallberg B.M., Gustafsson C.M., Larsson N.G. (2011). MTERF4 regulates translation by targeting the methyltransferase NSUN4 to the mammalian mitochondrial ribosome. Cell Metab..

[104] Spåhr H., Habermann B., Gustafsson C.M., Larsson N.-G., Hallberg B.M. (2012). Structure of the human MTERF4-NSUN4 protein complex that regulates mitochondrial ribosome biogenesis. Proc. Natl. Acad. Sci. U. S. A..

[105] Yakubovskaya E., Guja K.E., Mejia E., Castano S., Hambardjieva E., Choi W.S., Garcia-Diaz M. (2012). Structure of the essential MTERF4:NSUN4 protein complex reveals how an MTERF protein collaborates to facilitate rRNA modification. Structure.

[106] Scarpulla R.C. (2008). Transcriptional paradigms in mammalian mitochondrial biogenesis and function. Physiol. Rev..

[107] Metodiev M.D., Lesko N., Park C.B., Camara Y., Shi Y., Wibom R., Hultenby K., Gustafsson C.M., Larsson N.G. (2009). Methylation of 12S rRNA is necessary for in vivo stability of the small subunit of the mammalian mitochondrial ribosome. Cell Metab..

[108] Gleyzer N., Vercauteren K., Scarpulla R.C. (2005). Control of mitochondrial transcription specificity factors (TFB1M and TFB2M) by nuclear respiratory factors (NRF-1 and NRF-2) and PGC-1 family coactivators. Mol. Cell. Biol..

[109] Poldermans B., Bakker H., Van Knippenberg P.H. (1980). Studies on the function of two adjacent N6,N6-dimethyladenosines near the 3′ end of 16S ribosomal RNA of Escherichia coli. IV. The effect of the methylgroups on ribosomal subunit interaction. Nucleic Acids Res..

[110] Schluenzen F., Takemoto C., Wilson D.N., Kaminishi T., Harms J.M., Hanawa-Suetsugu K., Szaflarski W., Kawazoe M., Shirouzu M., Nierhaus K.H., Yokoyama S., Fucini P. (2006). The antibiotic kasugamycin mimics mRNA nucleotides to destabilize tRNA binding and inhibit canonical translation initiation. Nat. Struct. Mol. Biol..

[111] Charldorp R.V., Verhoeven J.J., Knippenberg P.H.V., Haasnoot C.A.G., Hilbers C.W. (1982). A carbon-13 neclear magnetic resonance study of the 3′-terminus of 16S ribosomal RNA of *Escherichia coli* specifically labeled with carbon-13 in the methlgroups of the m26Am26A sequence. Nucleic Acids Res..

[112] Heus H.A., Formenoy L.J., Van Knippenberg P.H. (1990). Conformational and thermodynamic effects of naturally occurring base methylations in a ribosomal RNA hairpin of Bacillus stearothermophilus. Eur. J. Biochem..

[113] Boehringer D., O'Farrell H.C., Rife J.P., Ban N. (2012). Structural insights into methyltransferase KsgA function in 30S ribosomal subunit biogenesis. J. Biol. Chem..

[114] Helser T.L., Davies J.E., Dahlberg J.E. (1972). Mechanism of Kasugamycin resistance in *Escherichia coli*. Nat. New Biol..

[115] Rozanska A., Richter-Dennerlein R., Rorbach J., Gao F., Lewis R.J., Chrzanowska-Lightowlers Z.M., Lightowlers R.N. (2017). The human RNA-binding protein RBFA promotes the maturation of the mitochondrial ribosome. Biochem. J..

[116] Sharma M.R., Barat C., Wilson D.N., Booth T.M., Kawazoe M., Hori-Takemoto C., Shirouzu M., Yokoyama S., Fucini P., Agrawal R.K. (2005). Interaction of era with the 30S ribosomal subunit implications for 30S subunit assembly. Mol. Cell.

[117] Dennerlein S., Rozanska A., Wydro M., Chrzanowska-Lightowlers Z.M., Lightowlers R.N. (2010). Human ERAL1 is a mitochondrial RNA chaperone involved in the assembly of the 28S small mitochondrial ribosomal subunit. Biochem. J..

[118] Jühling F., Pütz J., Florentz C., Stadler P.F. (2012). Armless mitochondrial tRNAs in Enoplea (Nematoda). RNA Biol..

[119] Fender A., Gaudry A., Jühling F., Sissler M., Florentz C. (2012). Adaptation of aminoacylation identity rules to mammalian mitochondria. Biochimie.

[120] Sprinzl M., Horn C., Brown M., Ioudovitch A., Steinberg S. (1998). Compilation of tRNA sequences and sequences of tRNA genes. Nucleic Acids Res..

[121] Perret V., Garcia A., Grosjean H., Ebel J.-P., Florentz C., Giegé R. (1990). Relaxation of a transfer RNA specificity by removal of modified nucleotides. Nature.

[122] Harrington K.M., Nazarenko I.A., Dix D.B., Thompson R.C., Uhlenbeck O.C. (1993). In vitro analysis of translational rate and accuracy with an unmodified tRNA. Biochemistry.

[123] Helm M., Brulé H., Degoul F., Cepanec C., Leroux J.P., Giegé R., Florentz C. (1998). The presence of modified nucleotides is required for cloverleaf folding of a human mitochondrial tRNA. Nucleic Acids Res..

[124] Helm M., Brulé H., Friede D., Giegé R., Pütz D., Florentz C. (2000). Search for characteristic structural features of mammalian mitochondrial tRNAs. RNA.

[125] Watanabe Y., Kawai G., Yokogawa T., Hayashi N., Kumazawa Y., Ueda T., Nishikawa K., Hirao I., Miura K., Watanabe K. (1994). Higher-order structure of bovine mitochondrial tRNA(SerUGA): chemical modification and computer modeling. Nucleic Acids Res..

[126] Frazer-Abel A.A., Hagerman P.J. (2008). Core flexibility of a truncated metazoan mitochondrial tRNA. Nucleic Acids Res..

[127] Wakita K., Watanabe Y., Yokogawa T., Kumazawa Y., Nakamura S., Ueda T., Watanabe K., Nishikawa K. (1994). Higher-order structure of bovine mitochondrial tRNA(Phe) lacking the 'conserved' GG and T psi CG sequences as inferred by enzymatic and chemical probing. Nucleic Acids Res..

[128] Helm M., Giegé R., Florentz C. (1999). A Watson−Crick Base-pair-disrupting methyl group (m1A9) is sufficient for cloverleaf folding of human mitochondrial tRNA Lys. Biochemistry.

[129] Motorin Y., Helm M. (2010). tRNA stabilization by modified nucleotides. Biochemistry.

[130] Voigts-Hoffmann F., Hengesbach M., Kobitski A.Y., van Aerschot A., Herdewijn P., Nienhaus G.U., Helm M. (2007). A methyl group controls conformational equilibrium in human mitochondrial tRNA(Lys). J. Am. Chem. Soc..

[131] Vilardo E., Nachbagauer C., Buzet A., Taschner A., Holzmann J., Rossmanith W. (2012). A subcomplex of human mitochondrial RNase P is a bifunctional methyltransferase--extensive moonlighting in mitochondrial tRNA biogenesis. Nucleic Acids Res..

[132] Richter U., Evans M.E., Clark W.C., Marttinen P., Shoubridge E.A., Suomalainen A., Wredenberg A., Wedell A., Pan T., Battersby B.J. (2018). RNA modification landscape of the human mitochondrial tRNA(Lys) regulates protein synthesis. Nat. Commun..

[133] Agris P.F. (1996). The importance of being modified: roles of modified nucleosides and Mg2+ in RNA structure and function. Prog. Nucleic Acid Res. Mol. Biol..

[134] Kawarada L., Suzuki T., Ohira T., Hirata S., Miyauchi K., Suzuki T. (2017). ALKBH1 is an RNA dioxygenase responsible for cytoplasmic and mitochondrial tRNA modifications. Nucleic Acids Res..

[135] Dewe J.M., Fuller B.L., Lentini J.M., Kellner S.M., Fu D. (2017). TRMT1-catalyzed tRNA modifications are required for redox homeostasis to ensure proper cellular proliferation and oxidative stress survival. Mol. Cell. Biol..

[136] Smith J.D., Dunn D.B. (1959). The occurrence of methylated guanines in ribonucleic acids from several sources. Biochem. J..

[137] Steinberg S., Cedergren R. (1995). A correlation between N2-dimethylguanosine presence and alternate tRNA conformers. RNA.

[138] Sonawane K.D., Bavi R.S., Sambhare S.B., Fandilolu P.M. (2016). Comparative structural dynamics of tRNA(Phe) with respect to hinge region methylated guanosine: a computational approach. Cell Biochem. Biophys..

[139] Jones C.I., Spencer A.C., Hsu J.L., Spremulli L.L., Martinis S.A., Derider M., Agris P.F. (2006). A counterintuitive Mg2+-dependent and modification-assisted functional folding of mitochondrial tRNAs. J. Mol. Biol..

[140] Dao V., Guenther R.H., Agris P.F. (1992). The role of 5-methylcytidine in the anticodon arm of yeast tRNA(Phe): site-specific Mg2+ binding and coupled conformational transition in DNA analogs. Biochemistry.

[141] Chen Y., Sierzputowska-Gracz H., Guenther R., Everett K., Agris P.F. (1993). 5-Methylcytidine is required for cooperative binding of Mg2+ and a conformational transition at the anticodon stem-loop of yeast phenylalanine tRNA. Biochemistry.

[142] Nobles K.N., Yarian C.S., Liu G., Guenther R.H., Agris P.F. (2002). Highly conserved modified nucleosides influence Mg2+-dependent tRNA folding. Nucleic Acids Res..

[143] Williams A.A., Darwanto A., Theruvathu J.A., Burdzy A., Neidigh J.W., Sowers L.C. (2009). Impact of sugar pucker on base pair and mispair stability. Biochemistry.

[144] Wakeman C.A., Winkler W.C. (2009). Analysis of the RNA backbone: structural analysis of riboswitches by in-line probing and selective 2′-hydroxyl acylation and primer extension. MIMB, Riboswitches.

[145] Newby M.I., Greenbaum N.L. (2002). Investigation of Overhauser effects between pseudouridine and water protons in RNA helices. Proc. Natl. Acad. Sci..

[146] Patton J.R., Bykhovskaya Y., Mengesha E., Bertolotto C., Fischel-Ghodsian N. (2005). Mitochondrial myopathy and sideroblastic anemia (MLASA): missense mutation in the pseudouridine synthase 1 (PUS1) gene is associated with the loss of tRNA pseudouridylation. J. Biol. Chem..

[147] Sundaralingam M., Rao S.T., Abola J. (1971). Molecular conformation of dihydrouridine: puckered base nucleoside of transfer RNA. Science (New York, N.Y.).

[148] Shigi N., Suzuki T., Terada T., Shirouzu M., Yokoyama S., Watanabe K. (2006). Temperature-dependent biosynthesis of 2-thioribothymidine of Thermus thermophilus tRNA. J. Biol. Chem..

[149] Grosjean H., Oshima T. (2007). Physiology and Biochemistry of Extremophiles.

[150] Dalluge J.J., Hamamoto T., Horikoshi K., Morita R.Y., Stetter K.O., McCloskey J.A. (1997). Posttranscriptional modification of tRNA in psychrophilic bacteria. J. Bacteriol..

[151] Rogalski M., Karcher D., Bock R. (2008). Superwobbling facilitates translation with reduced tRNA sets. Nat. Struct. Mol. Biol..

[152] Grosjean H., Westhof E. (2016). An integrated, structure- and energy-based view of the genetic code. Nucleic Acids Res..

[153] Johansson M.J.O., Esberg A., Huang B., Björk G.R., Byström A.S. (2008). Eukaryotic wobble uridine modifications promote a functionally redundant decoding system. Mol. Cell. Biol..

[154] Villarroya M., Prado S., Esteve J.M., Soriano M.A., Aguado C., Perez-Martinez D., Martinez-Ferrandis J.I., Yim L., Victor V.M., Cebolla E., Montaner A., Knecht E., Armengod M.-E. (2008). Characterization of human GTPBP3, a GTP-binding protein involved in mitochondrial tRNA modification. Mol. Cell. Biol..

[155] Li X., Li R., Lin X., Guan M.-X. (2002). Isolation and characterization of the putative nuclear modifier gene MTO1 involved in the pathogenesis of deafness-associated mitochondrial 12 S rRNA A1555G mutation. J. Biol. Chem..

[156] Yan Q., Bykhovskaya Y., Li R., Mengesha E., Shohat M., Estivill X., Fischel-Ghodsian N., Guan M.-X. (2006). Human TRMU encoding the mitochondrial 5-methylaminomethyl-2-thiouridylate-methyltransferase is a putative nuclear modifier gene for the phenotypic expression of the deafness-associated 12S rRNA mutations. Biochem. Biophys. Res. Commun..

[157] Yokoyama S., Watanabe T., Murao K., Ishikura H., Yamaizumi Z., Nishimura S., Miyazawa T. (1985). Molecular mechanism of codon recognition by tRNA species with modified uridine in the first position of the anticodon. Proc. Natl. Acad. Sci. U. S. A..

[158] Kirino Y., Yasukawa T., Ohta S., Akira S., Ishihara K., Watanabe K., Suzuki T. (2004). Codon-specific translational defect caused by a wobble modification deficiency in mutant tRNA from a human mitochondrial disease. Proc. Natl. Acad. Sci. U. S. A..

[159] Mayer C., Stortchevoi A., Köhrer C., Varshney U., Rajbhandary U.L. (2001). Initiator tRNA and its role in initiation of protein synthesis. Cold Spring Harb. Symp. Quant. Biol..

[160] Bilbille Y., Gustilo E.M., Harris K.A., Jones C.N., Lusic H., Kaiser R.J., Delaney M.O., Spremulli L.L., Deiters A., Agris P.F. (2011). The human mitochondrial tRNAMet: structure/function relationship of a unique modification in the decoding of unconventional codons. J. Mol. Biol..

[161] Lusic H., Gustilo E.M., Vendeix F.A.P., Kaiser R., Delaney M.O., Graham W.D., Moye V.A., Cantara W.A., Agris P.F., Deiters A. (2008). Synthesis and investigation of the 5-formylcytidine modified, anticodon stem and loop of the human mitochondrial tRNAMet. Nucleic Acids Res..

[162] Takemoto C., Spremulli L.L., Benkowski L.A., Ueda T., Yokogawa T., Watanabe K. (2009). Unconventional decoding of the AUA codon as methionine by mitochondrial tRNAMet with the anticodon f5CAU as revealed with a mitochondrial in vitro translation system. Nucleic Acids Res..

[163] Haag S., Sloan K.E., Ranjan N., Warda A.S., Kretschmer J., Blessing C., Hübner B., Seikowski J., Dennerlein S., Rehling P., Rodnina M.V., Höbartner C., Bohnsack M.T. (2016). NSUN3 and ABH1 modify the wobble position of mt-tRNAMet to expand codon recognition in mitochondrial translation. EMBO J..

[164] Van Haute L., Powell C.A., Minczuk M. (2017). Dealing with an unconventional genetic code in mitochondria: the biogenesis and pathogenic defects of the 5-formylcytosine modification in mitochondrial tRNA(met). Biomol. Ther..

[165] Boland C., Hayes P., Santa-Maria I., Nishimura S., Kelly V.P. (2009). Queuosine formation in eukaryotic tRNA occurs via a mitochondria-localized heteromeric transglycosylase. J. Biol. Chem..

[166] Chen Y.-C., Kelly V.P., Stachura S.V., Garcia G.A. (2010). Characterization of the human tRNA-guanine transglycosylase: confirmation of the heterodimeric subunit structure. RNA.

[167] Morris R.C., Brown K.G., Elliott M.S. (1999). The effect of queuosine on tRNA structure and function. J. Biomol. Struct. Dyn..

[168] Jenner L.B., Demeshkina N., Yusupova G., Yusupov M. (2010). Structural aspects of messenger RNA reading frame maintenance by the ribosome. Nat. Struct. Mol. Biol..

[169] Yarham J.W., Lamichhane T.N., Pyle A., Mattijssen S., Baruffini E., Bruni F., Donnini C., Vassilev A., He L., Blakely E.L., Griffin H., Santibanez-Koref M., Bindoff L.A., Ferrero I., Chinnery P.F., McFarland R., Maraia R.J., Taylor R.W. (2014). Defective i6A37 modification of mitochondrial and cytosolic tRNAs results from pathogenic mutations in TRIT1 and its substrate tRNA. PLoS Genet..

[170] Reiter V., Matschkal D.M.S., Wagner M., Globisch D., Kneuttinger A.C., Müller M., Carell T. (2012). The CDK5 repressor CDK5RAP1 is a methylthiotransferase acting on nuclear and mitochondrial RNA. Nucleic Acids Res..

[171] Lamichhane T.N., Blewett N.H., Crawford A.K., Cherkasova V.A., Iben J.R., Begley T.J., Farabaugh P.J., Maraia R.J. (2013). Lack of tRNA modification isopentenyl-A37 alters mRNA decoding and causes metabolic deficiencies in fission yeast. Mol. Cell. Biol..

[172] Stuart J.W., Gdaniec Z., Guenther R., Marszalek M., Sochacka E., Malkiewicz A., Agris P.F. (2000). Functional anticodon architecture of human tRNALys3 includes disruption of intraloop hydrogen bonding by the naturally occurring amino acid modification, t6A. Biochemistry.

[173] Lin H., Miyauchi K., Harada T., Okita R., Takeshita E., Komaki H., Fujioka K., Yagasaki H., Goto Y.I., Yanaka K., Nakagawa S., Sakaguchi Y., Suzuki T. (2018). CO_2_-sensitive tRNA modification associated with human mitochondrial disease. Nat. Commun..

[174] Urbonavicius J., Qian Q., Durand J., Hagervall T., Björk G.R. (2001). Improvement of reading frame maintenance is a common function for several tRNA modifications. EMBO J..

[175] Urbonavicius J., Stahl G., Durand J.M.B., Ben Salem S.N., Qian Q., Farabaugh P.J., Björk G.R. (2003). Transfer RNA modifications that alter +1 frameshifting in general fail to affect −1 frameshifting. RNA.

[176] Li J.-n., Esberg B., Curran J.F., Björk G.R. (1997). Three modified nucleosides present in the anticodon stem and loop influence the in vivo aa-tRNA selection in a tRNA-dependent manner. J. Mol. Biol..

[177] Hagervall T.G., Ericson J.U., Esberg K.B., Ji-nong L., Björk G.R. (1990). Role of tRNA modification in translational fidelity. Biochim. Biophys. Acta (BBA) - Gene Struct. Expr..

[178] Putz J., Florentz C., Benseler F., Giege R. (1994). A single methyl group prevents the mischarging of a tRNA. Nat. Struct. Biol..

[179] Zhang C.-M., Liu C., Slater S., Hou Y.-M. (2008). Aminoacylation of tRNA with phosphoserine for synthesis of cysteinyl-tRNA(Cys). Nat. Struct. Mol. Biol..

[180] Mohan A., Whyte S., Wang X., Nashimoto M., Levinger L. (1999). The 3′ end CCA of mature tRNA is an antideterminant for eukaryotic 3′-tRNase. RNA.

[181] Wilusz J.E., Whipple J.M., Phizicky E.M., Sharp P.A. (2011). tRNAs marked with CCACCA are targeted for degradation. Science (New York, N.Y.).

[182] Rorbach J., Nicholls T.J., Minczuk M. (2011). PDE12 removes mitochondrial RNA poly(a) tails and controls translation in human mitochondria. Nucleic Acids Res..

[183] Cooley L., Appel B., Soll D. (1982). Post-transcriptional nucleotide addition is responsible for the formation of the 5′ terminus of histidine tRNA. Proc. Natl. Acad. Sci. U. S. A..

[184] Nameki N., Asahara H., Shimizu M., Okada N., Himeno H. (1995). Identity elements of Saccharomyces cerevisiae tRNA(His). Nucleic Acids Res..

[185] Burkard U., Willis I., Soll D. (1988). Processing of histidine transfer RNA precursors. Abnormal cleavage site for RNase P. J. Biol. Chem..

[186] Preston M.A., Phizicky E.M. (2010). The requirement for the highly conserved G-1 residue of *Saccharomyces cerevisiae* tRNAHis can be circumvented by overexpression of tRNAHis and its synthetase. RNA.

[187] Nakamura A., Wang D., Komatsu Y. (2018). Biochemical analysis of human tRNA(His) guanylyltransferase in mitochondrial tRNA(His) maturation. Biochem. Biophys. Res. Commun..

[188] Giegé R., Sissler M., Florentz C. (1998). Universal rules and idiosyncratic features in tRNA identity. Nucleic Acids Res..

[189] Giegé R., Florentz C., Kern D., Gangloff J., Eriani G., Moras D. (1996). Aspartate identity of transfer RNAs. Biochimie.

[190] Fender A., Sauter C., Messmer M., Putz J., Giege R., Florentz C., Sissler M. (2006). Loss of a primordial identity element for a mammalian mitochondrial aminoacylation system. J. Biol. Chem..

[191] Tolkunova E., Park H., Xia J., King M.P., Davidson E. (2000). The human Lysyl-tRNA synthetase gene encodes both the cytoplasmic and mitochondrial enzymes by means of an unusual alternative splicing of the primary transcript. J. Biol. Chem..

[192] Mudge S.J., Williams J.H., Eyre H.J., Sutherland G.R., Cowan P.J., Power D.A. (1998). Complex organisation of the 5′-end of the human glycine tRNA synthetase gene. Gene.

[193] Nagao A., Suzuki T., Katoh T., Sakaguchi Y., Suzuki T. (2009). Biogenesis of glutaminyl-mt tRNAGln in human mitochondria. Proc. Natl. Acad. Sci..

[194] Friederich M.W., Powell C.A., Dallabona C., Kurolap A., Palacios-Zambrano S., Fernández-Moreno M.A., Baris H.N., Donnini C., Minczuk M., Rodenburg R.J., Van Hove J.L. (2018). Pathogenic variants in glutamyl-tRNAGln amidotransferase subunits cause a lethal mitochondrial cardiomyopathy disorder. Nat. Commun..

[195] Takeuchi N., Ueda T., Watanabe K. (1998). Expression and characterization of bovine mitochondrial methionyl-tRNA transformylase. J. Biochem..

[196] Rorbach J., Bobrowicz A., Pearce S., Minczuk M. (2014). Polyadenylation in bacteria and organelles. Methods Mol. Biol..

[197] Carlile T.M., Rojas-Duran M.F., Zinshteyn B., Shin H., Bartoli K.M., Gilbert W.V. (2014). Pseudouridine profiling reveals regulated mRNA pseudouridylation in yeast and human cells. Nature.

[198] Li X., Zhu P., Ma S., Song J., Bai J., Sun F., Yi C. (2015). Chemical pulldown reveals dynamic pseudouridylation of the mammalian transcriptome. Nat. Chem. Biol..

[199] Schwartz S., Bernstein D.A., Mumbach M.R., Jovanovic M., Herbst R.H., Leon-Ricardo B.X., Engreitz J.M., Guttman M., Satija R., Lander E.S., Fink G., Regev A. (2014). Transcriptome-wide mapping reveals widespread dynamic-regulated pseudouridylation of ncRNA and mRNA. Cell.

[200] Lovejoy A.F., Riordan D.P., Brown P.O. (2014). Transcriptome-wide mapping of pseudouridines: pseudouridine synthases modify specific mRNAs in S. cerevisiae. PLoS One.

[201] Li X., Xiong X., Zhang M., Wang K., Chen Y., Zhou J., Mao Y., Lv J., Yi D., Chen X.W., Wang C., Qian S.B., Yi C. (2017). Base-resolution mapping reveals distinct m(1)A methylome in nuclear- and mitochondrial-encoded transcripts. Mol. Cell.

[202] Safra M., Sas-Chen A., Nir R., Winkler R., Nachshon A., Bar-Yaacov D., Erlacher M., Rossmanith W., Stern-Ginossar N., Schwartz S. (2017). The m1A landscape on cytosolic and mitochondrial mRNA at single-base resolution. Nature.

[203] Hauenschild R., Tserovski L., Schmid K., Thuring K., Winz M.L., Sharma S., Entian K.D., Wacheul L., Lafontaine D.L., Anderson J., Alfonzo J., Hildebrandt A., Jaschke A., Motorin Y., Helm M. (2015). The reverse transcription signature of N-1-methyladenosine in RNA-Seq is sequence dependent. Nucleic Acids Res..

[204] Schwartz S. (2018). M1A within cytoplasmic mRNAs at single nucleotide resolution: a reconciled transcriptome-wide map. RNA.

[205] Natchiar S.K., Myasnikov A.G., Kratzat H., Hazemann I., Klaholz B.P. (2017). Visualization of chemical modifications in the human 80S ribosome structure. Nature.

[206] Morscher R.J., Ducker G.S., Li S.H., Mayer J.A., Gitai Z., Sperl W., Rabinowitz J.D. (2018). Mitochondrial translation requires folate-dependent tRNA methylation. Nature.

[207] Lightowlers R.N. (2011). Mitochondrial transformation: time for concerted action. EMBO Rep..

[208] Gammage P.A., Moraes C.T., Minczuk M. (2018). Mitochondrial genome engineering: the revolution may not be CRISPR-Ized. Trends Genet..

[209] Arenz S., Bock L.V., Graf M., Innis C.A., Beckmann R., Grubmuller H., Vaiana A.C., Wilson D.N. (2016). A combined cryo-EM and molecular dynamics approach reveals the mechanism of ErmBL-mediated translation arrest. Nat. Commun..

